# Tree peony seed oil alleviates hyperlipidemia and hyperglycemia by modulating gut microbiota and metabolites in high‐fat diet mice

**DOI:** 10.1002/fsn3.4108

**Published:** 2024-04-04

**Authors:** Ziyue Liang, Yinglong He, Dongfeng Wei, Peixin Fu, Yuying Li, Hao Wang, Di Yang, Xiaogai Hou

**Affiliations:** ^1^ College of Agriculture/Tree Peony Henan University of Science and Technology Luoyang China; ^2^ Key Laboratory of Efficient Cultivation and Comprehensive Utilization of Tree Peony in Henan Province Luoyang China; ^3^ College of Urban Construction, Luoyang Vocational and Technical College Luoyang China

**Keywords:** 16S rRNA, gut microbiota, high‐fat diet, metabolomics, tree peony seed oil

## Abstract

With the changes of people's lifestyle, hyperlipidemia and hyperglycemia which were induced from a diet high in both fat and sugar have become serious health concerns. Tree peony seed oil (PSO) is a novel kind of edible oil that shows great potential in the food industry because of its high constituent of unsaturated fatty acids. Based 16S rRNA and gut untargeted metabolomics, this study elucidated that the mechanism of PSO regulating blood glucose (Glu) and lipids. The impact of PSO on gut microbiota balance and gut metabolites of mice with a high‐fat diet (HFD) was evaluated. The findings indicated that PSO decreased HFD mice's body weight and fat accumulation, ameliorating the levels of blood lipid, reduced liver fat vacuole levels. What's more PSO modulated the proportion of gut microbiota in HFD mice and enhanced the abundance of probiotics. Furthermore, untargeted metabolomic analysis revealed that PSO not only impacted the generation of short‐chain fatty acids (SCFAs) by gut microorganism and altered metabolic pathway but exerted influence on secondary bile acids (BA), amino acid metabolism, and various other metabolites. These results suggested that PSO has the potential function for mitigating HFD‐induced hyperlipidemia and hyperglycemia by regulating gut microbiota and host metabolism.

## Graphical abstract

Tree peony seed oil (PSO) has the capacity of regulating gut microbiota and metabolites to decrease the body weights, fat vacuole levels triglyceride, total cholesterol, blood glucose, and low‐density lipoprotein. Our findings will allow readers to understand the mechanism of tree peony seed oil improving hyperlipidemia and hyperglycemia (By Figdraw).

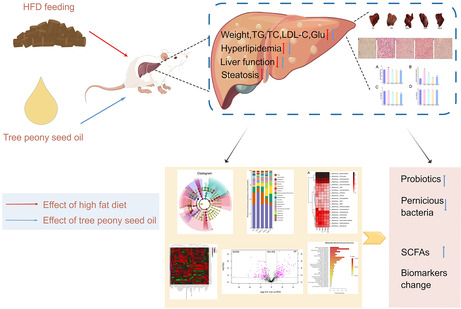

## 1 INTRODUCTION

Hyperlipidemia and hyperglycemia are common chronic disease often caused by lifestyle and diet, which is deemed to be related to all sorts of illnesses including hypertension, diabetes, obesity, overweight, and so on (Wang et al., 2019). Recent studies indicate that edible oils, abundant in unsaturated fatty acids, contribute to improving hyperlipidemia and obesity while also reducing plasma cholesterol levels (Kwek et al., 2022; Li et al., 2020). Woody edible oils, in particular, are noteworthy for their high content of both monounsaturated and polyunsaturated fatty acids, which could decrease serum triglyceride and avoid a variety of metabolic disorders (Tang et al., 2022; Wang et al., 2023). For instance, chlorella, blended oil, and other foods rich in unsaturated fatty acids possess the capabilities of improving obesity, lipid metabolism, and modulate gut microbiota in high‐fat diet (HFD) animal models (Chen et al., 2023; Yang, Ge, et al., 2022). Thus, tree peony seed oil (PSO) is a perspective natural food rich in unsaturated fatty acids that can be employed for the improvement of hyperlipidemia and lipid metabolism disorders.

Long‐term intake of a lot of sugar and fat can lead to intestinal flora imbalance and change the dominant flora (Guo et al., 2021; Jia et al., 2013; Yue et al., 2020). For instance, Zhou team's research showed that the Bacteroidetes and *Lactobacillus* had a decline of abundance in HFD‐induced mice, but the Proteobacteria's abundance increased (Zhou et al., 2023). Recent years, some studies have shown that hyperglycemia can lead to gut barrier dysfunction and increase the hazard of enteric infection, which provide more opportunities for bacteria to enter the body (Cynthia & Bernd, 2023; Thaiss et al., 2018).

PSO has been favored by consumers in recent years because it has plenty of unsaturated fatty acids, comprising oleic acid (OA), alpha‐linolenic acid (ALA), and linoleic acid (LA) (Deng et al., 2022; He, Wang, Li, et al., 2023). Some studies have shown that PSO exhibits the oxidative stability, antimicrobial activities, and ameliorates neuroinflammation‐mediated cognitive deficits (He, Wang, Zhao, et al., 2023; Jie et al., 2021; Bai et al., 2021). In addition, foundational research has substantiated the ability of PSO to lower blood lipid and blood glucose (Glu) levels, employing diverse methodologies ranging from chemical assays to animal models. Multiple studies have reported that PSO may contribute to alleviating elevated blood lipid and blood sugar due to its excellent anti‐obesity effect (Dong et al., 2013; Yuan et al., 2019). Su team's research stated that the hypolipidemic effect of PSO operates by inhibiting lipogenesis and enhancing fatty acid β‐oxidation (Su et al., 2016). Wang et al. (2022) claimed that PSO is efficacious in hyperglycemia and diabetes mellitus in vitro/vivo. However, the mechanism of PSO for improving hyperlipidemia and hyperglycaemia has not yet been elucidated.

The mouse model induced by a HFD to mimic hyperlipidemia and hyperglycemia exhibits behavior similar to that of humans, providing a valuable approach for studying the pathogenesis of these conditions (Yang, Zhong, et al., 2022). Therefore, in the present study, hyperlipemia and hyperglycemia in mice induced by HFD were deployed to explore the protective effect of PSO. Gut microbic sequencing and liquid chromatography‐mass spectrometry (LC–MS) were harnessed to delve deeper into the underlying mechanism of PSO in bringing down hyperlipemia and hyperglycemia, which lowered the Glu and blood lipids by regulating gut microbes and metabolites. The results offer a theoretical foundation for further expanding the application of PSO for hyperlipidemia.

## 2 MATERIALS AND METHODS

### 2.1 Samples and chemicals

The PSO sample used in this experiment was purchased from the Luoyang Tianjiao Tree Peony Technology Co., Ltd (Henan, China). Chemical standard undecanotriglyceride triglyceride internal standard solution was bought from Sigma‐Aldrich (St. Louis, Missouri, USA).

### 2.2 Analysis of the constituents of PSO

Analysis of constituents in PSO was done by gas chromatograph mass spectrometer (GC–MS, Agilent Technologies Co., LTD, Santa Clara, California, USA). Using a DB‐WAX flexible quartz capillary column, PSO underwent methyl esterification, employing triglyceride undecanoate as an internal standard. Chromatographic column: HP‐5MS: 30 m × 0.25 mm × 0.25 um; Column flow: 0.8 mL/min; Column temperature: initial temperature 80°C. The temperature was raised to 250°C at a rate of 8°C/min. Carrier gas: high‐purity helium; Injection port temperature: 250°C; Injection volume: 1 μL; Injection method: split 20:1; GC–MS interface temperature: 250°C; EI source: (70 eV), ion source: 230°C, quadrupole temperature: 150°C, EM voltage: 1.294 V, scan range: 27 ~ 460 amu. A variety of unsaturated fatty acids were detected in PSO. These compounds were identified on the basis of the exact characteristic ion (Lin et al., 2021; Zhang et al., 2021).

### 2.3 Animal experiments and dosage information

A number of fifty SPF grade male ICR mice, aged 6 weeks with a weight of about 30 g, were purchased from Henan SKobes Biotechnology Co., LTD (Henan, China; License number SCXK2020‐0005). The temperature range was maintained at 25 ± 2°C with a relative humidity of 40 ± 5%, following a 12‐h light–dark cycle, clean bedding, and ad libitum access to both water and standard dry pellet feed. All protocols in this study were approved by the Animal Experiment Committee of Henan University of Science and Technology (No. 20190719016). All animal experiments were performed in accordance with the American Veterinary Medical Association (AVMA) Guidelines for the Euthanasia of Animals (2020). The standard forage included 18 to 22% protein, 9% water, 4% fat, 8% ash, 5% fiber, and 52 to 56% nitride‐free extracts. The HFD consisted of 20% lard, 15% sucrose, 1.5% cholesterol, 0.15% sodium cholate, and the remainder fed conventionally. Following a one‐week period for adaptation, the mice were randomly assigned to the basal diet control group (Con group, *n* = 10) and the HFD group (*n* = 40). Except for the normal mice, all high‐fat model mice were fed with HFD for 8 weeks. Throughout the study, the control group of mice consistently received the same water. After 8 weeks, the high‐fat mice were divided into high, medium, and low doses of PSO. The treatment groups were structured as follows: (1) control group: fed with 1.5 mL normal saline + basal diet. (2) PSO low‐dose group (PSO‐L group): fed with 0.5 mL PSO + 1 mL normal saline + basal diet. (3) PSO medium‐dose group (PSO‐M group): fed with 1 mL PSO + 0.5 mL normal saline. (4) PSO high‐dose group (PSO‐H group): fed with 1.5 mL PSO + basal diet for 4 weeks (Aldamarany et al., 2023; Chen et al., 2023).

### 2.4 Sample collection and measurement

During the course of the experiment, mice were weighed weekly, and at the last day, mouse feces were collected. Also, the heart was punctured to take blood, and the serum was taken by centrifugation at 3000 rpm for 15 minutes. For biochemical analysis, livers were collected from sacrificed mice. Serum total cholesterol (TC), triglyceride (TG), high‐density lipoprotein cholesterol (HDL‐C) and low‐density lipoprotein cholesterol (LDL‐C), and Glu were analyzed by commercial diagnostic kits (Nanjing Jiancheng BioEngineering Institute, Nanjing, China). Moreover, mice body weight, epididymal fat weight, liver weight, and body length (from nose apex to anus) were measured to count epididymal fat index, liver index, and Lee's efficiency (Sun et al., 2019).

(1) $$\text{Liver index}=\frac{\text{Tissue weight}}{\text{Body mass weight}\;}\times 100\%$$

(2) $${\mathrm{Lee}}^{\prime }s\;\text{efficiency}=\sqrt[{3}]{\text{final body weight}\;\left(g\right)\times 10^{3}/\text{body length}\;\left(\mathrm{cm}\right)\;}$$

### 2.5 Histologic analysis

Mouse liver tissues underwent overnight fixation in 4% paraformaldehyde, followed by dehydration using gradient ethanol. They were subsequently embedded in paraffin, sectioned, and stained with hematoxylin and eosin (HE) before examination under a Nikon Eclipse ci microscope.

### 2.6 Gut microbiota profiling by 16S rRNA sequencing

The analysis of gut microbiota composition involved extracting total genomic DNA from the samples. DNA purity was assessed on a 1% agarose gel, and the concentration was adjusted to 1 ng/μL with sterile water. Subsequently, a sequencing adapter was attached to the end of the primer after designing conserved region primers and performing PCR amplification. Purified PCR products were used to prepare sequencing libraries.

The raw reads attained from the sequencing were percolated using Trimmomatic v 0.33 software. Usearch v10 software was utilized to merge the clean reads from each sample, and the merged data length was percolated based on the corresponding length ranges for different regions. The chimeric sequences were detected and subsequently removed using UCHIME v4.2 software to obtain the final set of valid reads. Usearch software was employed to cluster the sequences with a similarity threshold of 97.0%, resulting in operational taxonomic units (OTUs). SILVA was utilized as the reference database for taxonomic annotation of the feature sequences. QIIME 2 software (v 1.8.0) was used to select the sequences of the most abundant features at the taxonomic level of the phylum and genus as representative sequences. QIIME 2 was used to assess the α‐diversity and β‐diversity indices of the samples.

### 2.7 Untargeted metabolome analysis of intestinal microbes

Fecal metabolites were isolated using a triple TOF‐6600 mass spectrometer (AB Sciex, USA) and LC20 ultra‐performance liquid chromatography (UPLC) with a Waters ACQUITY UPLC HSS T3 C18 column (100 mm × 2.1 mm, 1.8 μm, Shimadzu, USA). A temperature of 40°C was upheld for the column, and an injection volume of 2 μL was utilized. The mobile phases composed of ultrapure water with phase A (containing 0.1% formic acid) and acetonitrile with phase B (containing 0.1% formic acid) eluting at a flowing at a rate of 0.4 mL/min. The following parameters defined the gradient elution conditions: 0–11 min, 95%–10% phase A; 11–12.1 min, 10%–95% phase A; 12.1–14 min, 95% phase A; 0–11 min, 5%–90% phase B; 11–12 min, 90% phase B; 12.1–14 min, and 5% phase B.

In the LC–MS/MS analysis process, the positive and negative data were imported into the MetaboAnalyst R software package (v 3.1.3). Next, partial least squares discriminant analysis (PLS‐DA) and orthogonal partial least‐squares discriminant analysis (OPLS‐DA) were conducted to visualize the metabolic differences among experimental groups. The magnitude of metabolite change was measured by fold change (FC), combined with *P* values, and screen metabolites with significant differences between the groups.

### 2.8 Statistical analysis

SPSS software v 22.0 (SPSS, Inc., Chicago, IL, USA) was used for statistical analysis. One‐way ANOVA and least significant difference (LSD) multiple comparison tests were used for comparison between groups. The results were plotted using Origin 8.5 software.

## 3 RESULTS

### 3.1 Determination of fatty acid composition of PSO

GC–MS was employed to determine the composition and content of unsaturated fatty acids in PSO, which was shown in Table 1. Five components were identified by GC–MS, of which three most abundant were 9‐Octadecenoic acid, 9,12‐Octadecadienoic acid, and 9,12,15‐Octadecatrienoic acid. The contents of the three major components of PSO were further quantified, revealing that the contents in PSO were 24.03 ± 0.27%, 23.35 ± 0.25%, and 41.74 ± 0.74%, respectively.

**TABLE 1:** Principal component analysis of fatty acids of PSO.

No.	Compound	Relative content (%)
1	Hexadecanoic acid (palmitic acid)	6.04 ± 0.07
2	Stearate (stearic acid)	2.25 ± 0.04
3	9‐Octadecenoic acid (Z)‐(oleic acid)	24.03 ± 0.27
4	9,12‐Octadecadienoic acid (linoleic acid)	23.35 ± 0.25
5	9,12,15‐Octadecatrienoic acid (linolenic acid)	41.74 ± 0.74

### 3.2 Effects of PSO on physiology of HFD‐induced mice

**FIGURE 1:**
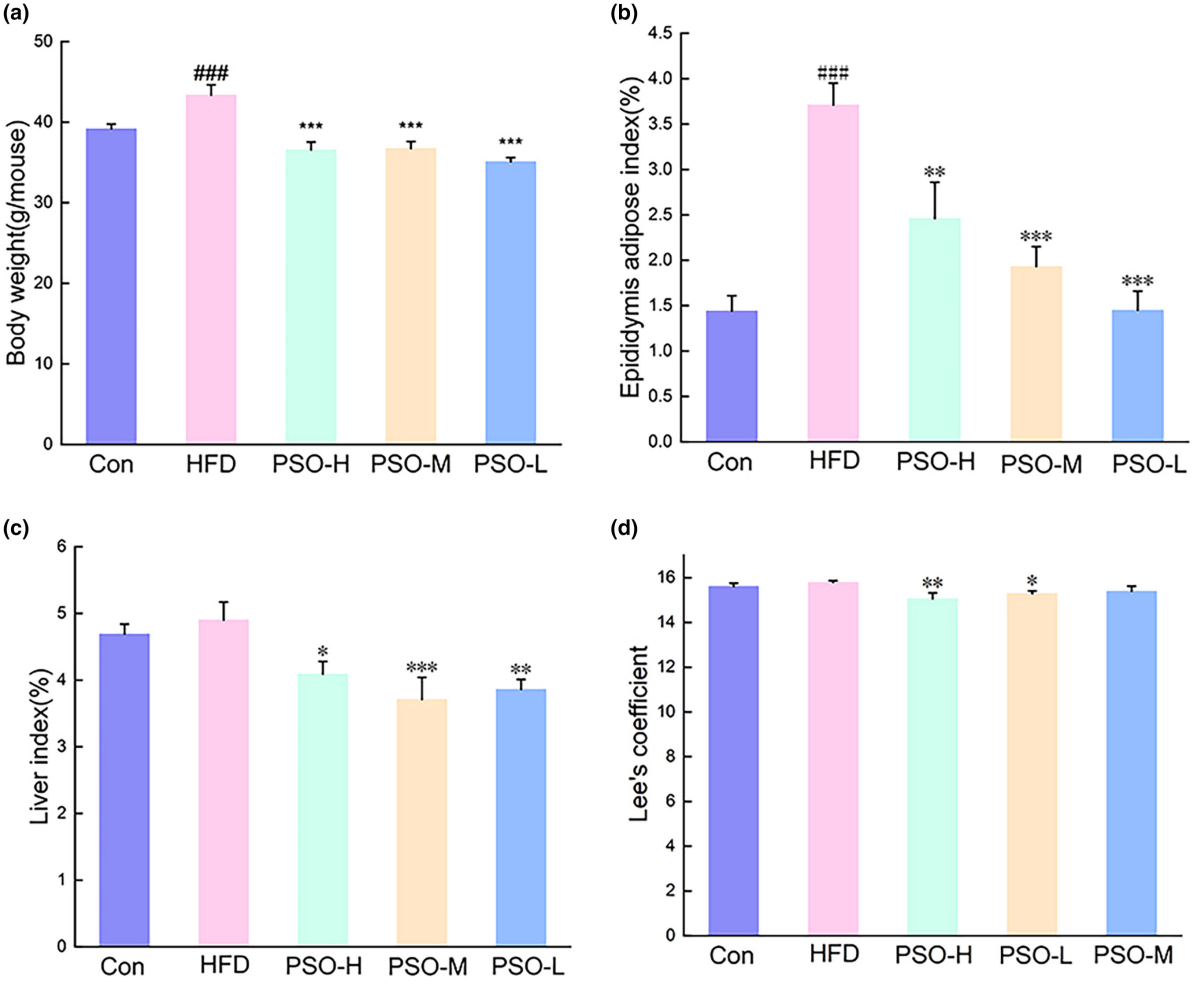
Effects of PSO on body weight gain, epididymis fat, liver weights, and Lee's coefficient induced by HFD. (a) Final body weight of mice in different groups. Effects of PSO on epididymis fat (b), liver weights (c), and Lee's coefficient (d). The data were presented as the mean ± SEM (*n* = 10). ^###^ *p <* .001, compared with that of the Con group, *p < .05, **p < .01, ***p < .001, compared with that of the HFD group.

The results showed that PSO (*p <* .001) could reduce the body weights of mice in HFD when compared with HFD group (Figure 1a). Compared with mice in the Con group, mice in the HFD group (*p <* .001) displayed a marked increase in epididymis adipose index in terms of fat weights (Figure 1b), while there was obvious decrease for epididymis adipose index in PSO‐H group (*p <* .01), PSO‐M group (*p <* .001), and PSO‐L group (*p <* .001) at the end of the experiment. After PSO administration for 4 weeks, liver indexes significantly decreased in PSO‐H group (*p <* .05), PSO‐M group (*p <* .001), and PSO‐L group (*p <* .01, Figure 1c). Moreover, Lee's coefficient was higher in mice from the HFD group compared to those in PSO‐H (*p <* .01) and PSO‐L group (*p <* .05, Figure 1d).

**FIGURE 2:**
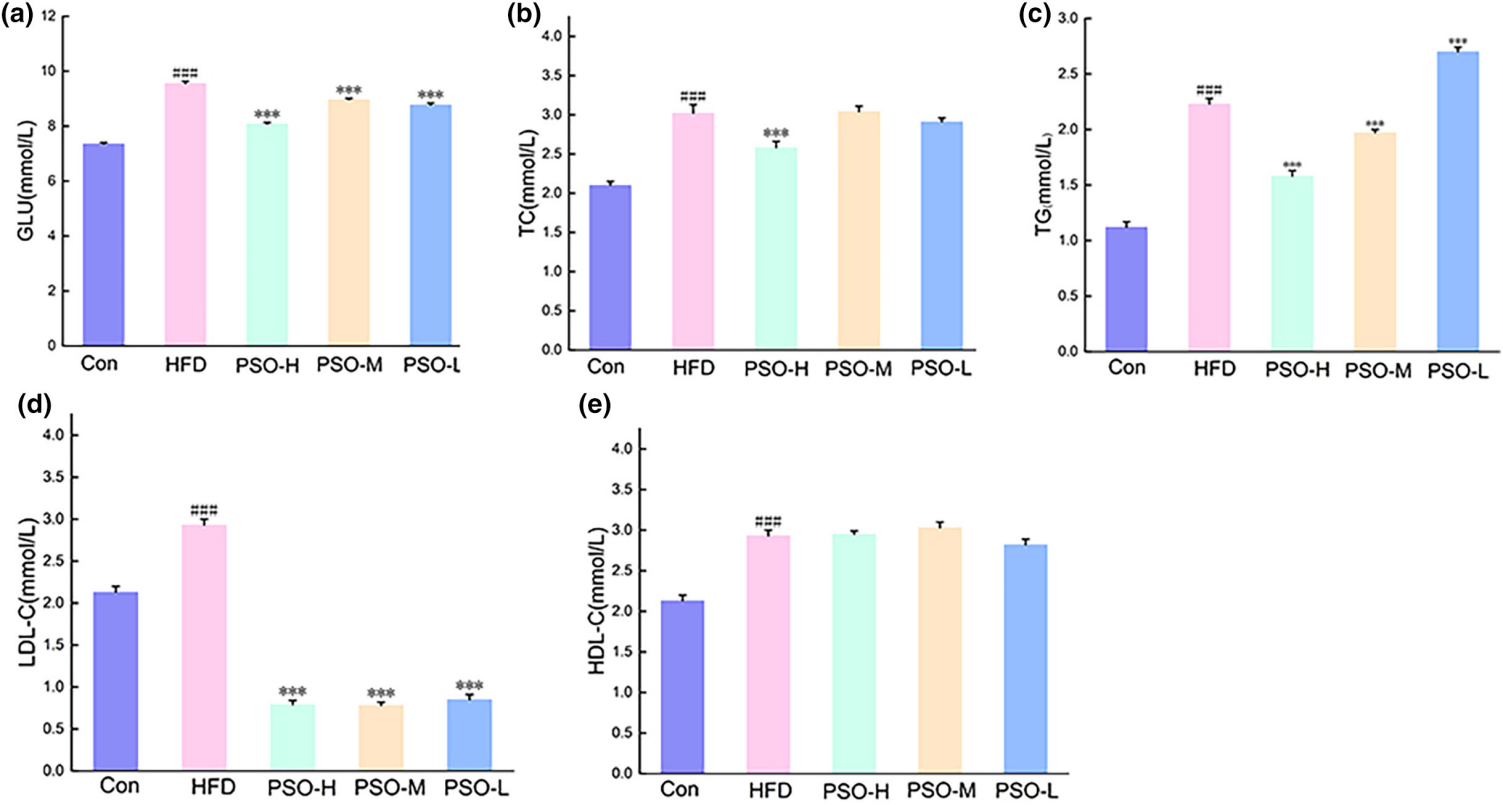
Effect of PSO on the levels of Glu (a), TG (b), TC (c), LDL (d), and HDL (e) in mice. The data were presented as the mean ± SEM (n = 10). ^###^ *p* < .001, compared with that of the Con group, ***p < .001, compared with that of the HFD group.

HFD‐induced obese mice may emerge abnormal lipid metabolism phenomenon. Thus, the study employed PSO to HFD‐induced mice to observe whether the PSO has the impact on serum lipid of HFD mice. On the one hand, compared with Con group, the level of Glu, TC, TG, LDL‐C, and HDL‐C of HFD group was markedly different (*p <* .001, Figure 2a–d). On the other hand, after a 4‐week intervention of PSO, the level of Glu, TC, TG, and LDL‐C of PSO group was down‐regulated significantly compared with HFD group (*p <* .001). But the level of HDL‐C had no marked difference among the groups (Figure 2e). As the result, studies have indicated that PSO has the capacity of ameliorating lipid metabolism disorders.

**FIGURE 3:**
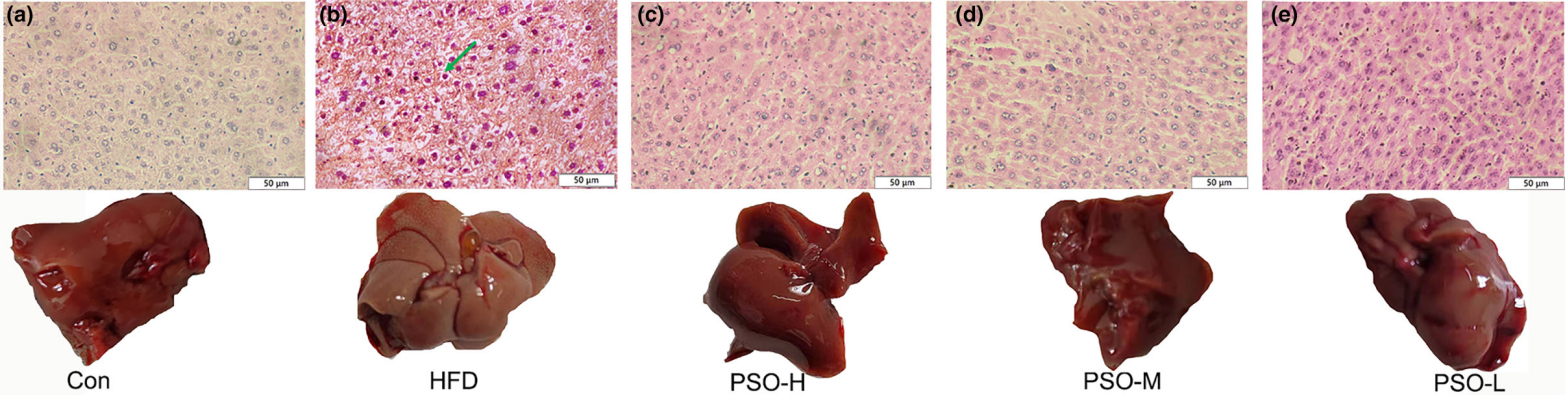
Representative images of hematoxylin and eosin‐stained liver tissue. (a): Con, (b): HFD, (c): PSO‐H, (d): PSO‐M, e: PSO‐L, the green arrow was tiny round fat vacuole, scale bar = 50 μm.

Fatty liver disease will be generated due to excess HFD. This study observed the characteristics of histology of mice livers to confirm that whether PSO had the competence of reducing accumulation of lipochondria. As shown in Figure 3a, it showed the the Con group mice liver. Compared with Con group, the liver tissue of HFD group shows visible pathological changes (Figure 3b). Also, fat vacuoles were obviously observed in the liver cytoplasm (green arrow), and there were obvious inflammatory changes in hepatocytes, indicating that the mice had severe non‐alcoholic fatty liver disease (NAFLD). After giving by PSO, as expected, the phenomenon of fat accumulation in liver tissues in PSO‐H group, PSO‐M group and PSO‐L group disappeared. (Figure 3c–e). Taken together, these findings underscored the alleviating impact of PSO in HFD mice.

### 3.3 Effects of PSO on gut microbiota composition

**FIGURE 4:**
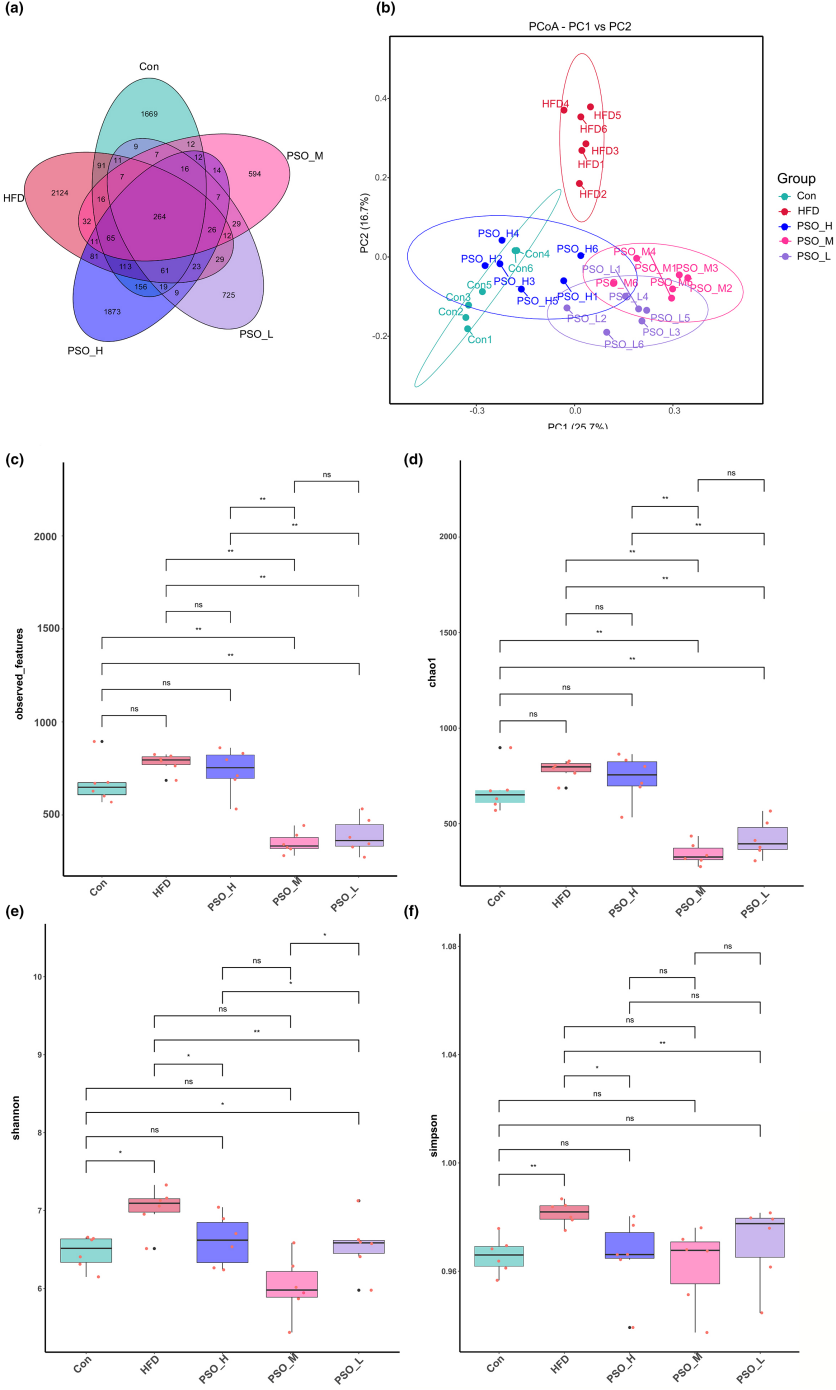
Effects of PSO on the gut microbiota alterations in HFD mice. (a) Venn graph showing the OTU numbers of gut microbiota from five groups. (b) PCoA analysis of the Bray–Curtis distance based on OTUs. Analysis of alpha‐diversity abundance Observed OTUs (c), Chao1 (d), Shannon (e), and Simpson (f) indices in each group. ns: no significant difference, *p < .05, **p < .01.

The gut microorganisms of mice were assessed through 16S rRNA amplicon sequencing to investigate the alleviative effect of PSO on HFD‐induced mice, and a total of 8117 OTUs were found in 30 samples based on 97% sequence similarity (Table S1). Among them, 2528 OTUs were in Con group, 2966 OTUs were in HFD group, 2750 OTUs were in PSO‐H group, 1124 OTUs were in PSO‐M group, and 1254 OTUs were in PSO‐L group. Of these, 264 OTUs were common to the five groups. And there were 1669, 2124, 1837, 594, and 725 OTUs specific to the Con, HFD, PSO‐H, PSO‐M, and PSO‐L groups, respectively (Figure 4a). Principal coordinate analysis results (PCoA) of the Bray–Curtis distances showed HFD was significantly separated from Con, PSO‐H, PSO‐M, and PSO‐L. Also, crossover clustering was observed between PSO‐H and Con (Figure 4b). The results of the observed features and Chao1 indexes based on alpha diversity analysis demonstrated that PSO‐M and PSO‐L reversed the dysregulation (*p <* .01, Figure 4c,d). In addition, Shannon (*p <* .05) and Simpson (*p <* .01) indexes showed the species richness and evenness of HFD group had a significant decrease compared with Con group. While PSO‐H (*p <* .05) and PSO‐M (*p <* .01), influenced community diversity showing PSO‐H and PSO‐M enriched the gut microbiota abundance of HFD mice (Figure 4e,f).

**FIGURE 5:**
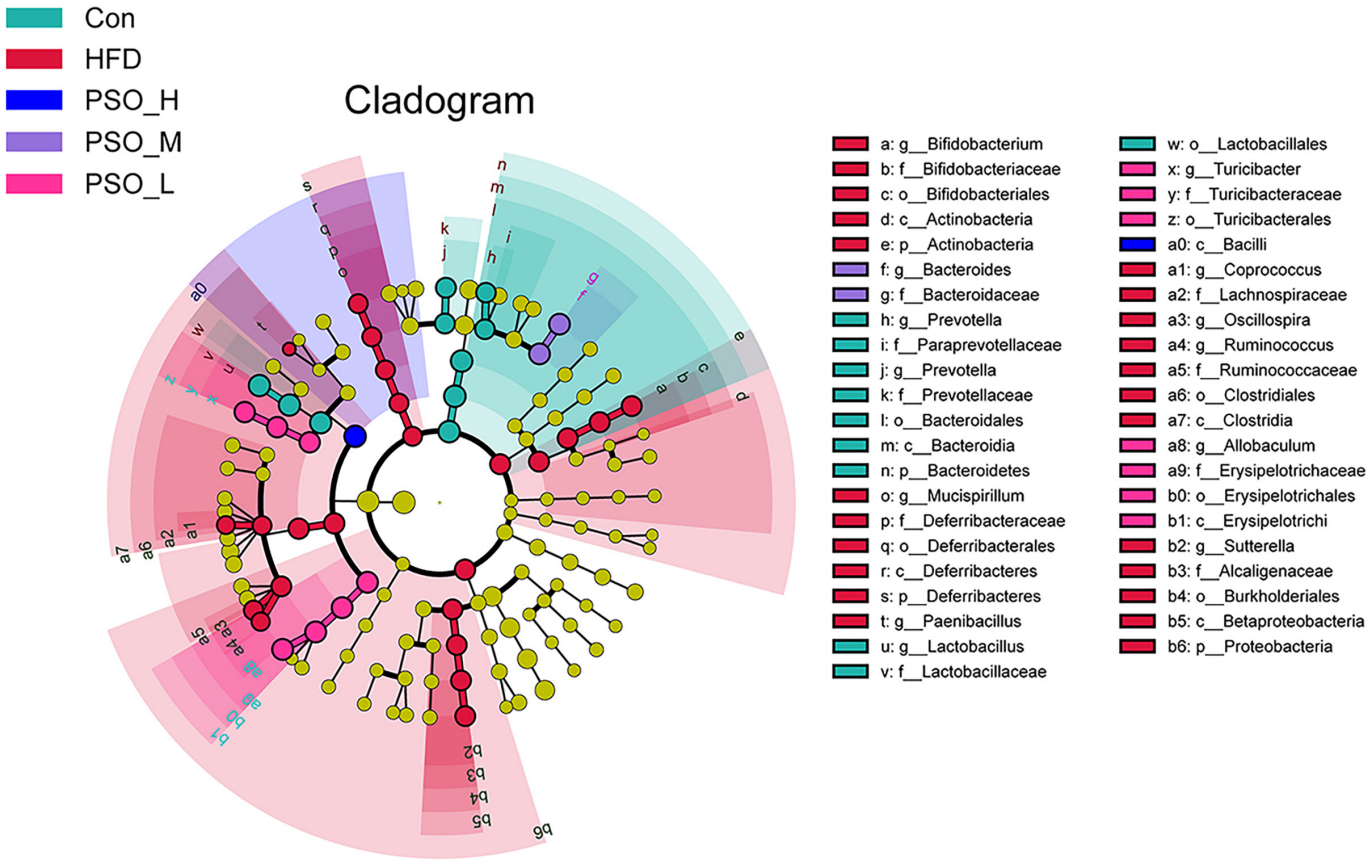
Cladogram plot from Lefse analysis among the different groups.

Min‐max normalization‐based taxa heatmap showed conspicuous species abundance differences among 5 groups (Figure S1a,b). In the meanwhile, LDA was used to determine the significantly different species (*p <* .05, LDA > 4). At different taxonomic levels, Figure S1c presents the chosen relative abundances of the gut microbiota. The higher the LDA score, the greater is the relative prevalence of the corresponding group. Overall, the abundance of Con group and PSO groups was higher than that of the HFD group. Corresponding cladogram was used to identify the biological markers with statistical differences in different groups at genus level (Figure 5). There were significant effects on *Prevotella* and *Lactobacillus* in the Con group; *Bifidobacterium*, *Mucispirillum*, *Paenibacillus*, *Coprococcus*, *Oscillospira*, *Ruminococcus*, and *Sutterella* in the HFD group; *Bacteroides* in the PSO‐M group; And *Turicibacter* and *Allobaculum* in the PSO‐L group (*p <* .05) (Zhao et al., 2019).

**FIGURE 6:**
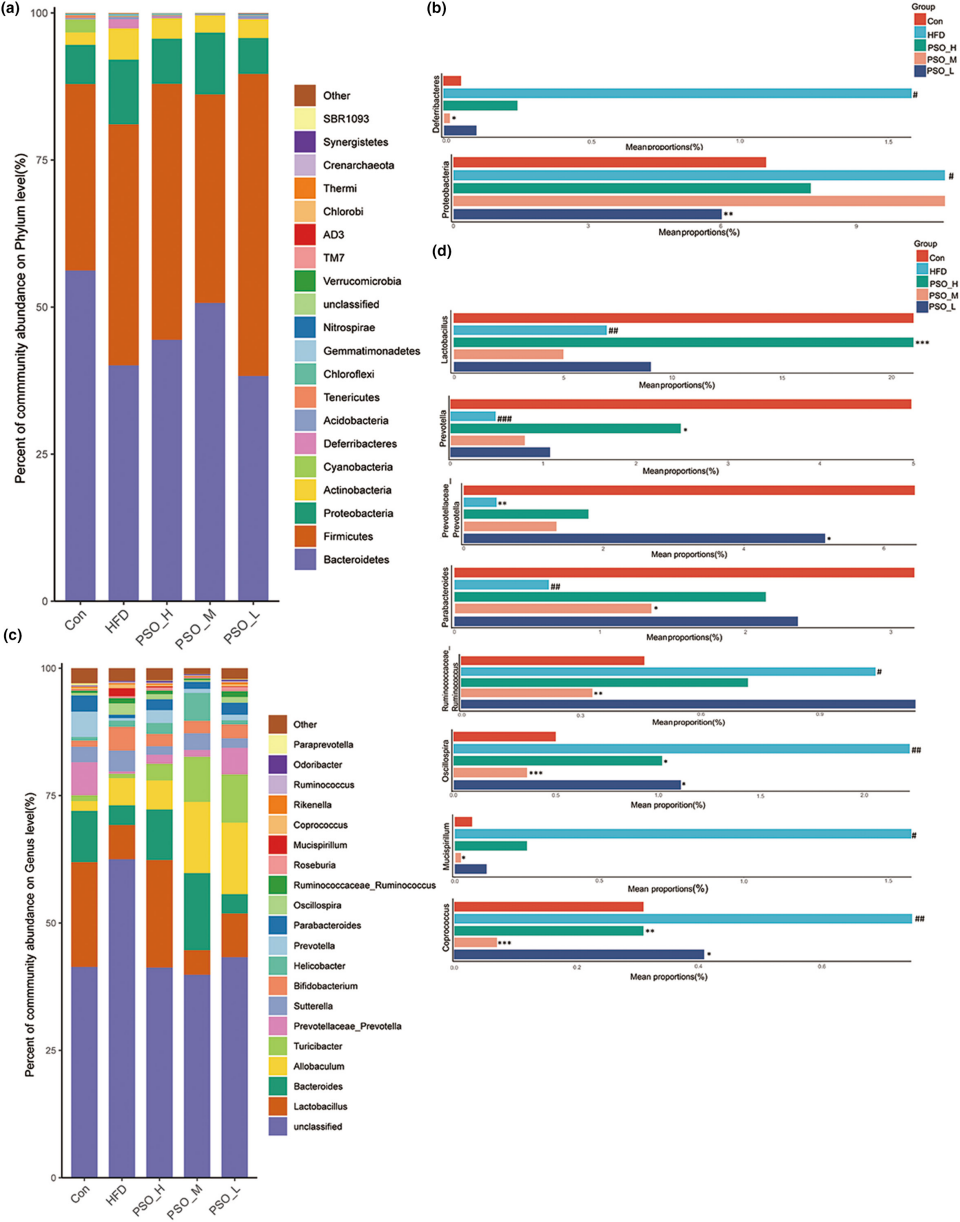
Impact of PSO on gut microbial structure in HFD mice. (a) Microbial community bar plot at phylum level (top 20). (b) Relative abundance of the significantly altered bacteria at the phylum levels from the five groups. (c) Microbial community bar plot at genus level (top 20). (d) Relative abundance of the significantly altered bacteria at the genus levels from the five groups. ^#^ *p <* .05, ^##^ *p <* .01, ^###^ *p <* .001, compared with that of the Con group, *p < .05, **p < .01, ***p < .001, compared with that of the HFD group.

Next, this study further analyzed the taxonomic distributions of microbial composition which were caused by HFD group and restored by PSO. The predominant bacterial of Con group was Bacteroidetes at phylum level, followed by Firmicutes and Proteobacteria. In HFD group, nevertheless, these predominant bacteria were Firmicutes, Bacteroidetes, and Proteobacteria in turn (Figure 6a). Compared with Con group, the Proteobacteria and Deferribacteres of HFD group presented marked difference (*p <* .05, Figure 6b). After 4 weeks of gavage using PSO, PSO‐L group decreased the abundance of Proteobacteria (*p <* .01) and PSO‐M lowered the abundance of Deferribacteres significantly (*p <* .05). Next, this study further analyzed the top 20 taxa at the genus level (Figure 6c). The identified bacteria in genus of mice include *Lactobacillus*, *Bacteroides*, *Prevotellaceae‐Prevotella*, *Sutterella*, and so on. Compared with the Con group, the abundance of probiotic *Lactobacillus* (*p <* .01), *Prevotellaceae_Prevotella* (*p <* .01), *Prevotella* (*p <* .001), and *Parabacteroides* (*p <* .01) was reduced in the HFD group (Figure 6d). In addition, the abundance of *Ruminococcaceae_Ruminococcus* (*p <* .05), *Oscillospira* (*p <* .01), *Mucispirillum* (*p <* .05), and *Coprococcus* (*p <* .01) that an excess of these microbiota was profitless were significantly increased. Noteworthily, PSO group reversed these trends of microbiota imbalance caused by HFD (Figure 6d). Compared with the HFD group, PSO‐H upregulated the proportions of *Lactobacillus* (*p <* .05) and *Prevotella* (*p <* .001), PSO‐M obviously elevated the presence of *Parabacteroides* and reducing the prevalence of *Ruminococcaceae_Ruminococcu* (*p <* .05) and *Mucispirillum* (*p <* .01), and PSO‐L significantly promoted the reduction of *Ruminococcaceae_Ruminococcu* (*p <* .05) (Figure 6d). Interestingly, *Oscillospira* (*p <* .05) and *Coprococcus* (*p <* .001) in all PSO group showed an obvious decrease (Figure 6d). These results indicated that PSO was capable of alleviating regulate the intestinal ratio and augment the probiotic species.

### 3.4 Effect of PSO on gut metabolic disorders in mice

**FIGURE 7:**
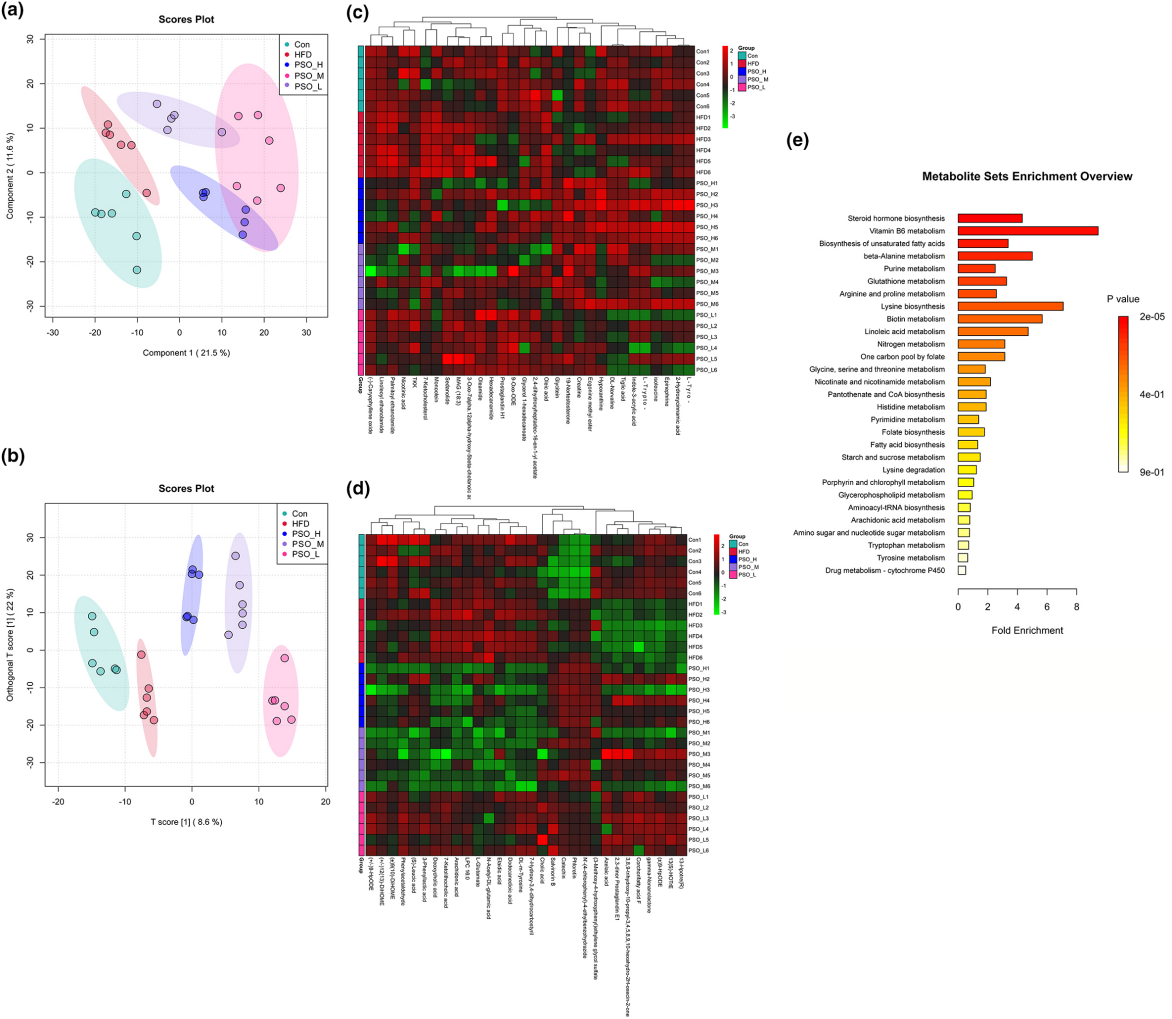
Effects of PSO on intestinal metabolites. (a) PLS‐DA score plot of Con, HFD, PSO‐H, PSO‐M, and PSO‐L groups in positive mode. (b) OPLS‐DA score plot from Con, HFD, PSO‐H, PSO‐M, and PSO‐L groups in positive mode. (c) Heatmap of the top 30 metabolites of 5 groups in positive mode. (d) Heatmap of the top 30 metabolites of 5 groups in negative mode. (e) Metabolic pathway enrichment analysis of PSO‐H vs HFD.

The distribution of low‐molecular weight metabolites in obese mice was evaluated by the untargeted metabolomics analysis. The results of PLS‐DA and OPLS‐DA scores plot manifested HFD groups were separated from Con group and three PSO groups had distinct separations in positive mode (Figure 7a,b). Permutation tests proved that the predictive ability of the model was remarkable (Figure S2a,b). And negative mode plot also exhibited analogous results, which indicated that PSO could regulate the metabolism of gut microbiota (Figure S3a,b). In addition, the levels of these 30 metabolites in the different experimental groups are shown in Figure 7c. According to the classification of HMDB, at positive levels, PSO intervention induced altered metabolites including organic acids and derivatives (5), phenylpropanoids and polyketides (2), benzenoids (1), organoheterocyclic compounds (5), lipids and lipid‐like molecules (10), alkaloids and derivatives (1), organic nitrogen compounds (1), and others (5, Table S2). At negative levels, 9 belonged to lipids and lipid‐like molecules, 2 belonged to organic acids and derivatives, 3 belonged to phenylpropanoids and polyketides, 1 belonged to benzenoids, and 15 belonged to others (Figure 7d, Table S3). The box plot also exhibited representative differential metabolites in which the ranking was the top (top 25 with lower P values; Figure S4). Among these metabolites, 2‐(acetylamino)‐3‐(1H‐indol‐3‐yl) propanoic acid and lysine butyrate belongs to SCFAs, and box plot displayed that the abundance of butyrate in PSO groups was higher than that in HFD group. Based on MetaboAnalyst tool, in the Con vs HFD group, the predominant three metabolic pathways were steroid hormone biosynthesis, vitamin B6 metabolism, histidine metabolism (Figure S5a). Besides, PSO‐H and PSO‐M significantly reversed the high‐fat diet‐induced changes in histidine metabolism (Figure 7e, Figure S5a). PSO‐L significantly reversed the fat diet‐induced changes in steroid hormone biosynthesis and vitamin B6 metabolism (Figure S5c). These results indicated the changes of PSO on gut metabolites.

### 3.5 Association analysis of serum lipid indicators and gut microbiota and metabolites

**FIGURE 8:**
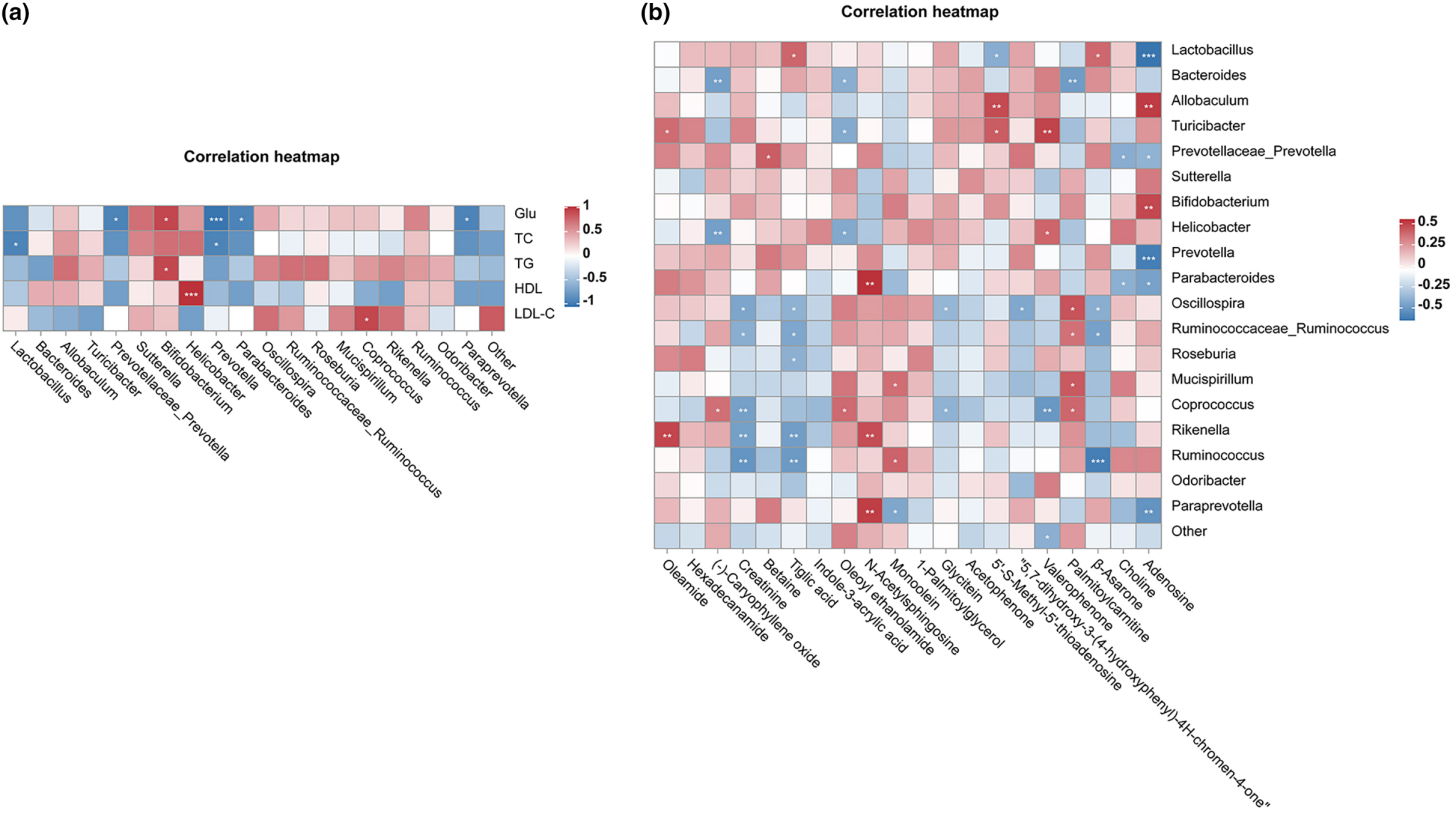
(a) Correlation heatmap between differential genus‐level bacteria and serum. (b) Correlation heatmap between differential genus‐level bacteria and differential metabolites (positive and negative ion combined) analysis. *p < .05, **p < .01, ***p < .001.

Correlations between metabolites, biochemical parameter, and metabolism were analyzed using the Spearman correlation. The study performed a correlation matrix linking the top 20 bacteria with the aforementioned 5 biochemical indexes. The biochemical levels of Glu (*p <* .05) and TG (*p <* .05) showed positive correlations with *Bifidobacterium* (Figure 8a). HDL‐C positively correlated with *Helicobacter* (*p <* .001). LDL‐C had a positive correlation with *Coprococcus* (*p <* .05). However, Glu presented significantly negatively correlated with *Paraprevotella* (*p <* .05), *Parabacteroides* (*p <* .05), *Prevotella* (*p <* .001), and *Prevotellaceae_Prevotella* (*p <* .05). TC showed highly marked negative *Prevotella* (*p <* .05) and *Lactobacillus* (*p <* .05). The association analysis of top 20 bacteria and metabolites found that 12 bacteria were positively correlated with 10 metabolites. On the other hand, the results revealed that 12 bacteria had a negative correlation with 12 metabolites (Figure 8b). Those descriptions manifested that gut microbiota played an essential role in regulating the microbial metabolism involved in the hyperlipidemia and hyperglycemia induced by HFD.

## 4 DISCUSSION

The metabolites which are generated through gut microbiota play significant parts in maintenance of a healthy balance in body. In the past years, a lot of studies have provedthat unsaturated fatty acids could regulate and alleviate dyslipidemia (Jing et al., 2022; Zong et al., 2020). On the basis of previous studies, we revealed the molecular mechanism of PSO in lowering Glu and lipids via gut microbiome and untargeted metabolomics approaches, delving the influences of PSO on level of serum lipid, gut microbiota, and metabolites, and their potential relation in HFD‐induced mice.

We affirmed that HFD led to lipid metabolism in disorder of mice, while PSO was a condition where there was a decrease in weight, TC, TG, Glu, and LDL‐C. This is in line with the effect of not only onion oil but also garlic oil on serum lipids and parameters of obesity (Chao et al., 2018). Some dietary supplements have been reported to prevent dyslipidemia by different mechanisms (Hunter & Hegele, 2017). As the consequence, these results demonstrated the improved effect of PSO.

Gut microbes as a crucial regulator of metabolic diseases are strongly associated with diabetes, hyperlipidemia and hyperglycemia, obesity, and NAFLD (Bai et al., 2023; Chi et al., 2019; Pitocco et al., 2020). In this study, we verified that PSO gavage had a significant influence on the gut microorganic composition of mice, which demonstrated gut microbiota exerted on the regulation of HFD‐induced hyperlipidemia and hyperglycemia. In gut bacteria, *Bacteroides* are known as probiotics and they are specific strains that metabolize cholesterol (Le et al., 2022). Whereas *Firmicutes* are positively correlated with obesity. The intervention of PSO enhanced the abundance of *Bacteroides* and reduced the abundance of *Firmicutes* in HFD‐induced mice. In addition, *Ruminococcuz* can increase in type 2 diabetes and obesity or overweight (Henke et al., 2019); A research has discovered *Mucispirillum* has a higher abundance in obese mice (Floch, 2017); *Oscillospira* has a positive correlation with the progression of type 2 diabetes constipation, and body weights (Feng et al., 2020; Konikoff & Gophna, 2016). The changes of *Coprococcus* are positively related with the changes of body weights, TG, and TC (Yarullina et al., 2020). While PSO decreased the abundance of these bacteria. These outcomes align with prior studies demonstrating that reshaping the balance of gut microbiota ameliorates hyperlipidemia and hyperglycemia (McGinley et al., 2020).

For SCFAs, indirect nutrients produced by intestinal microbiota usually have remarkable physiological regulation function. Butyric acid and propionic acid are involved in lipid metabolism and regulate intestinal PH. Besides, intestinal flora affects energy supply, Glu, and lipid homeostasis through butyrate, which can modulate physiological and pathological processes associated with obesity. Also, SCFAs may exert a protective role in autoimmune islet function and slow down the development of diabetes. Moreover, SCFAs have a regulatory effect on cardiovascular system (Jin et al., 2021). Significant differences in metabolites butyric acid and propionic acid were detected in our untargeted metabolomics assay. The results indicated that PSO could modulate SCFAs production to prevent hyperlipidemia and hyperglycemia.

Studies have shown that metabolomics reveals that microbial amino acid metabolism is involved in hyperlipemia (Yan et al., 2022). In accordance with other studies (Zhu et al., 2022), alterations of amino acid metabolism in HFD‐induced mice were observed in our study. These perturbed amino acids included threonine, isoleucine, tryptophan, etc. Among these amino acids, according to report, dietary threonine restriction may delay the development of obesity‐related metabolic dysfunction (Yap et al., 2020). In our untargeted metabolomics assay, the threonine content of mice in HFD group was higher, while the content of PSO group was lower than Con group. Branched‐chain amino acids emerge positively correlated with type 2 diabetes mellitus. The reduction of isoleucine can bring metabolic health benefits and its effects are more evident than valine (Yu et al., 2021). In our experiment, we found that isoleucine was lower in the PSO‐L group than in both the high‐fat and Con groups. Moreover, tryptophan is positively correlated with the risk of T2D (Qi et al., 2022). The abundance of tryptophan in HFD was higher than Con group. PSO‐M and PSO‐L reversed the increased trend, which indicated that regulation of amino acid metabolism was one of the mechanisms of PSO in the improvement of hyperlipemia.

Bile acid (BA) is not only involved in fat digestion and absorption and cholesterol metabolism but also plays an essential part in diabetes, obesity, NAFLD, and other metabolic diseases (Jia et al., 2021). Primary bile acids are secreted into the gut, where they are dehydroxylated to form secondary bile acids under the action of intestinal bacteria (Connors et al., 2020), such as taurodeoxycholic acid, aminodeoxycholic acid, deoxycholic acid, lithocholic acid, glycolithocholic acid, taurocholic acid, and so on. In our assay, high‐fat diet reduced bile acid levels of HFD group, but the levels of aminodeoxycholic acid and taurodeoxycholic acid were higher in HFD group than in Con group. The taurodeoxycholic acid of mice in PSO‐M and PSO‐L groups was decreased, which was consistent with previous studies (Ding et al., 2021). Deoxycholic acid and taurocholic acid can increase energy expenditure, being beneficial for losing weight. More deoxycholic acid and taurocholic acid were detected in the gut metabolites of PSO groups’ mice, and HFD group was lower than normal level. The intervention with PSO dose‐dependently altered the content of these BA, indicating that PSO has the potential to modulate bile acid production, thereby mitigating fat accumulation.

Based on serum level, relevant heat maps of intestinal flora and metabolites were made. There were differences in serum levels and intestinal flora, as well as intestinal flora and gut metabolites. The abundance of *Prevotella*, *Parabacteroides*, and *Coprococcus* was correlated with Glu, TC, and TG, which have been shown to be associated with obesity and diabetes (Christensen et al., 2019; Valentine et al., 2020; Wang et al., 2019). Intestinal microbiota carries out complex and active metabolic activities in the intestine, with the help of *Lactobacillus*, *Bacteroides*, and other strains, they can produce SCFAs, BA, choline metabolites, indoles derivatives, vitamins, etc. to regulate lipid metabolism, diet‐induced obesity, and diabetes.

In summary, PSO intervention markedly decreased HFD mice's body weight, fat accumulation, and ameliorate hyperlipidemia hyperglycemia by modulating gut microorganism, SCFAs, amino acid, and other intestinal metabolites. Hence, our findings suggest that PSO holds promise as a potential dietary intervention method for improving hyperlipidemia and hyperglycemia in future.

## 5 CONCLUSION

PSO is a prospective functional food, which could decrease the weight of HFD‐induced mice, improve blood lipid status, and inflammatory level of liver. Moreover, based on 16S rRNA sequencing results, we found that PSO could change the structure of intestinal flora, regulate intestinal metabolic disorders, and increase the abundance of probiotics. Untargeted metabolomics results showed that peony seed oil could regulate the metabolism of intestinal microbiota, increase the content of SCFAs, change metabolic pathways, reduce the levels of threonine, isoleucine and tryptophan, and regulate BA levels. Therefore, PSO exhibits potential as a dietary intervention strategy for improving hyperlipidemia and hyperglycemia.

## AUTHOR CONTRIBUTIONS

Conceptualization, X.H.; methodology, X.H. and Y.H.; software, Z.L., Y.H. and X.H.; validation, Z.L., Y.H. and X.H.; formal analysis, Z.L., Y.H. and P.F.; investigation, Z.L., H.W., Y.L. and D.Y.; resources, D.W; data curation, Z.L., Y.H. and X.H.; writing—original draft preparation, Z.L. and Y.H.; writing—review and editing, Z.L., Y.H., Y.L. and X.H; visualization, Z.L. and Y.H.; supervision, X.H.; project administration, X.H.; funding acquisition, X.H. All authors have read and agreed to the published version of the manuscript.

## CONFLICT OF INTEREST STATEMENT

The authors have no conflicts of interest.

## Supporting information


Figure S1.

Table S1.

## Data Availability

The data presented in this study are available on request from the corresponding author. Data sets generated and/or analyzed in the current study are available in supplementary information.
