# Supplementary material for: Tree peony seed oil alleviates hyperlipidemia and hyperglycemia by modulating gut microbiota and metabolites in high‐fat diet mice
Source: Food Sci Nutr. 2024 Apr 4;12(6):4421–34. doi: 10.1002/fsn3.4108 (PMC11167153; doi:10.1002/fsn3.4108)
Supplement: Supplementary file 1 — Figure S1. [file FSN3-12-4421-s001.docx]

**Tree peony seed oil alleviates hyperlipidemia and hyperglycemia by modulating gut microbiota and metabolites in high-fat diet mice**

**Supplementary Figures**

**
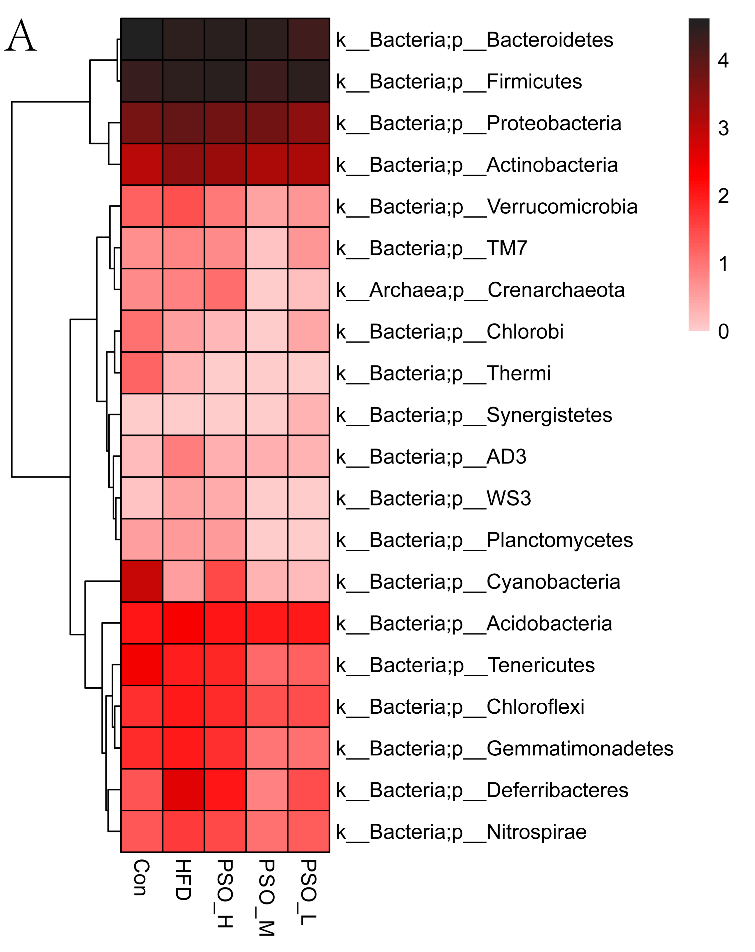
**

**
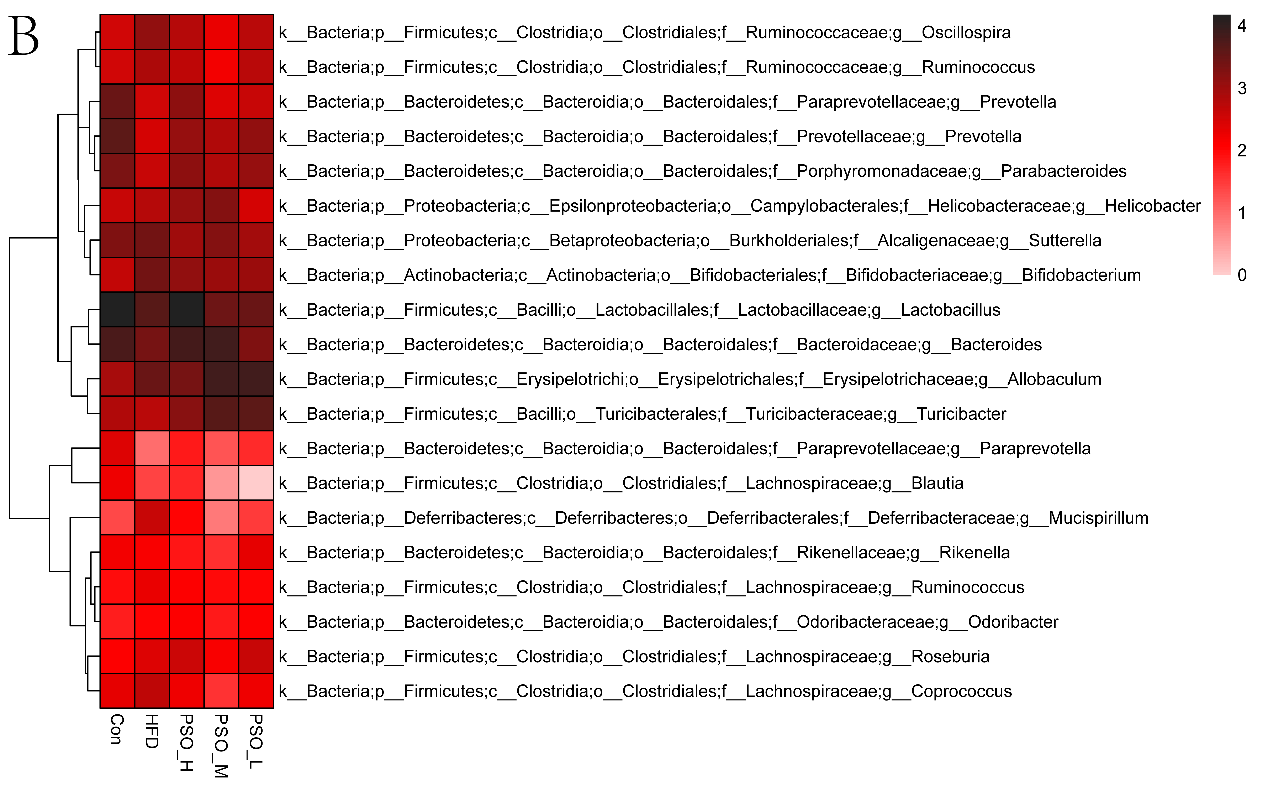
**


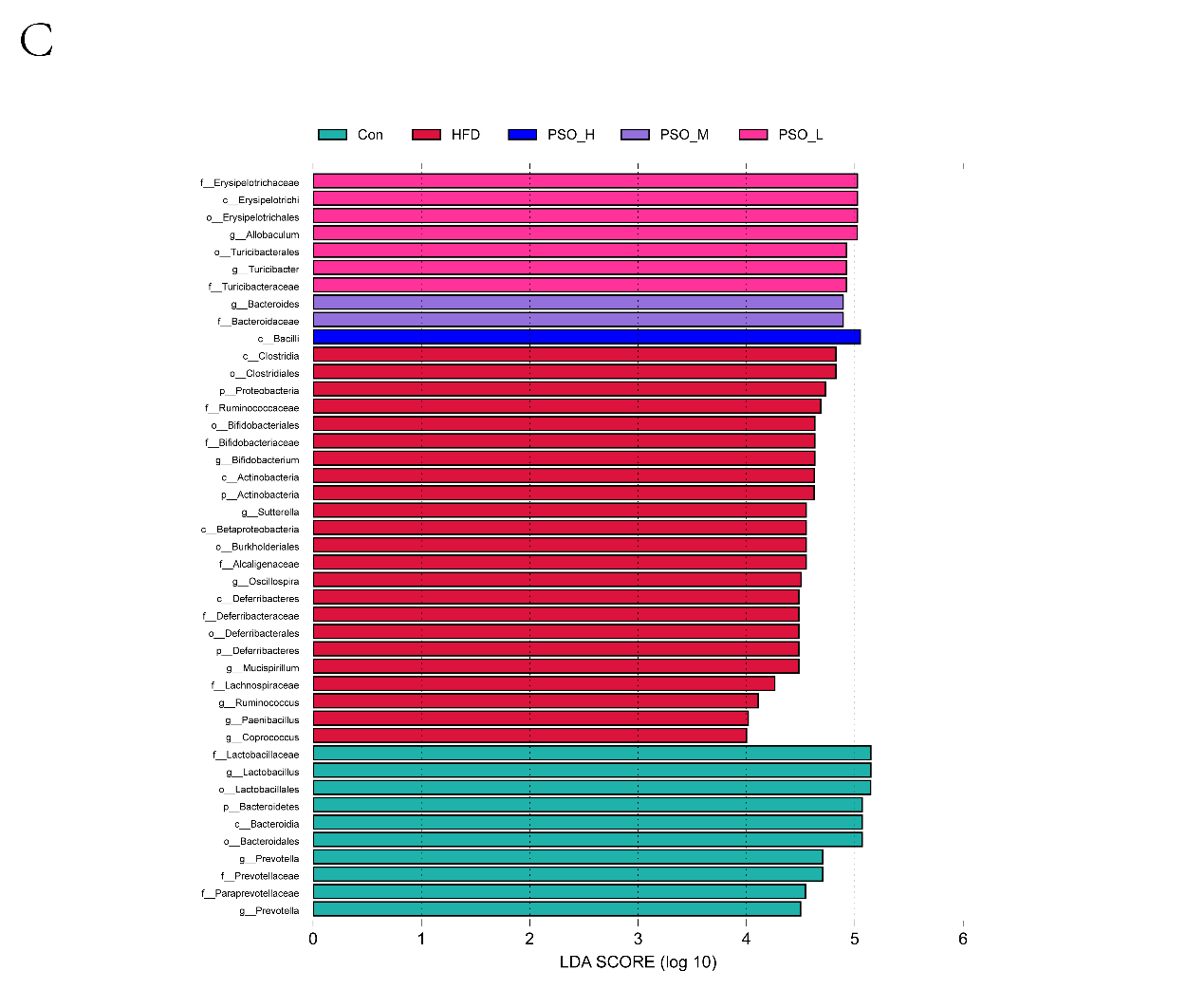


**Figure S1.** Relative abundance of microorganisms at phylum (A) and (B) genus level. The values in two heatmaps correspond to standardized Min-max normalization generated from taxa abundance and (C) showing the differences of bacterial abundance were generated from LEfSe analysis.


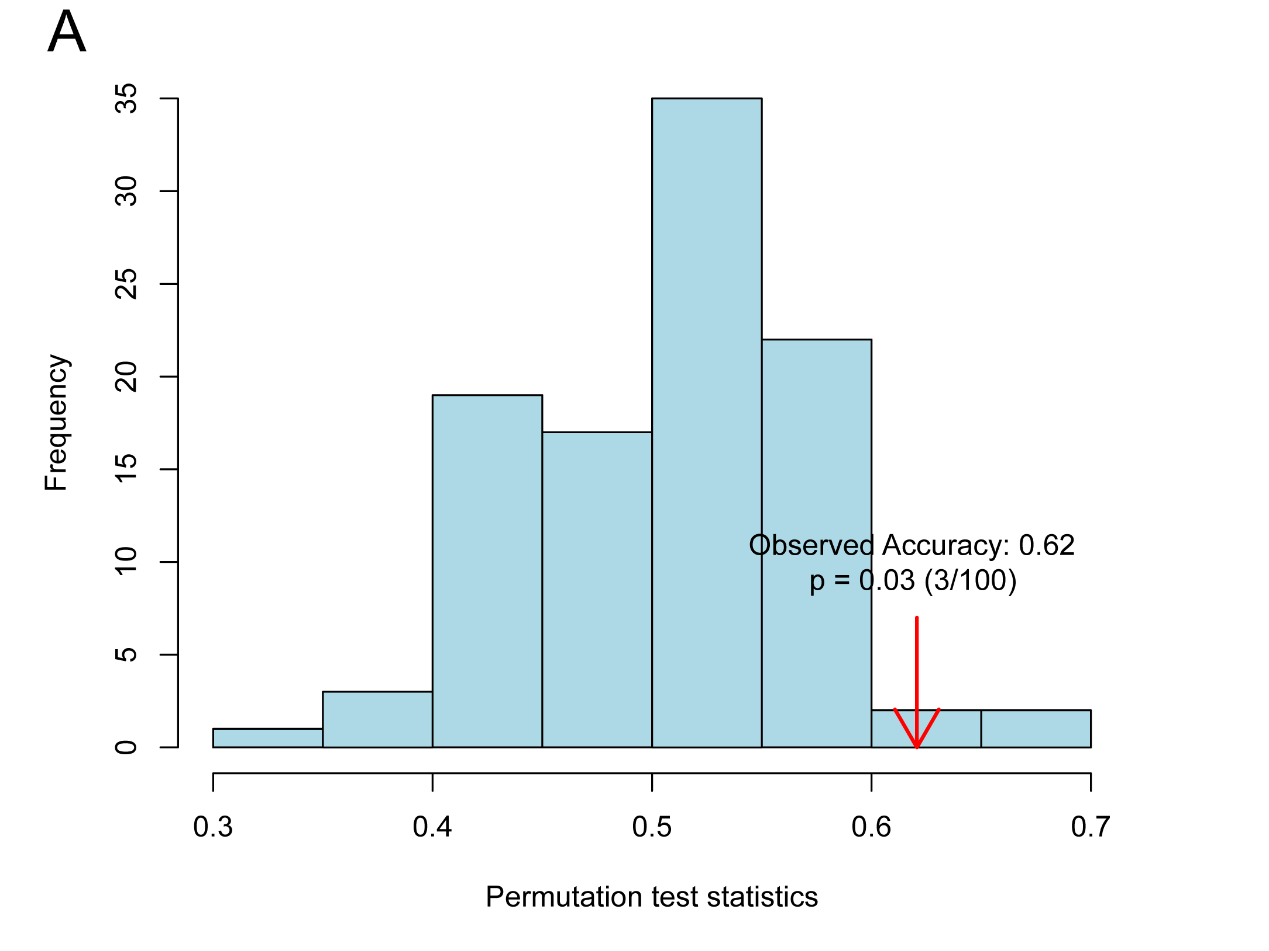


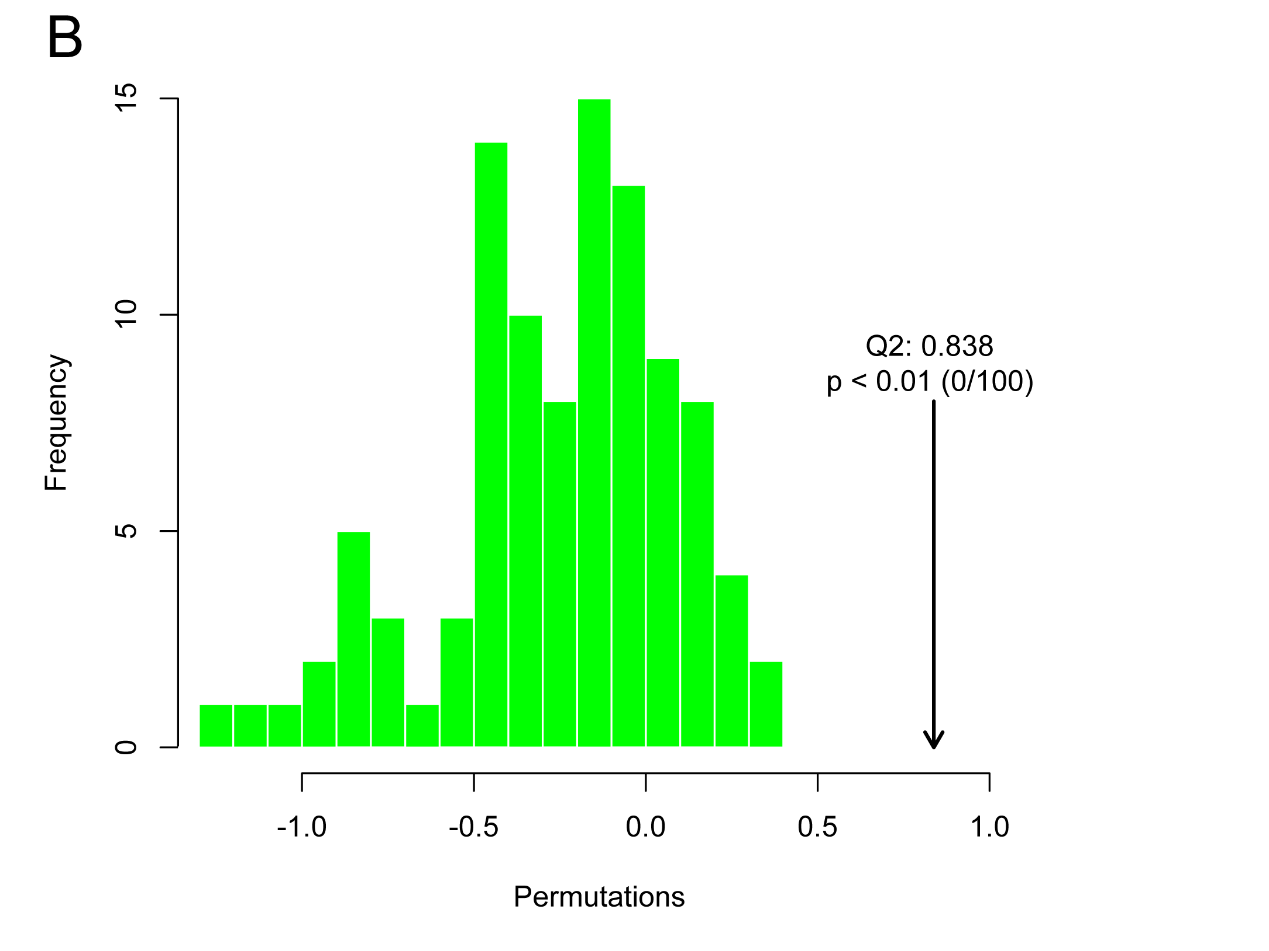


Figure S2. Permutation test in positive mode (A) Distribution of test statistics for PLS-DA permutation test. (B) Test statistics (Q2) distribution of OPLS-DA permutation test.


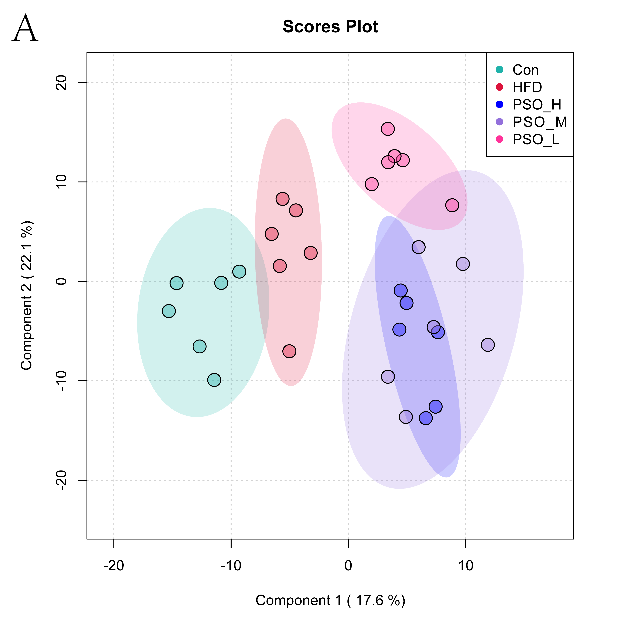

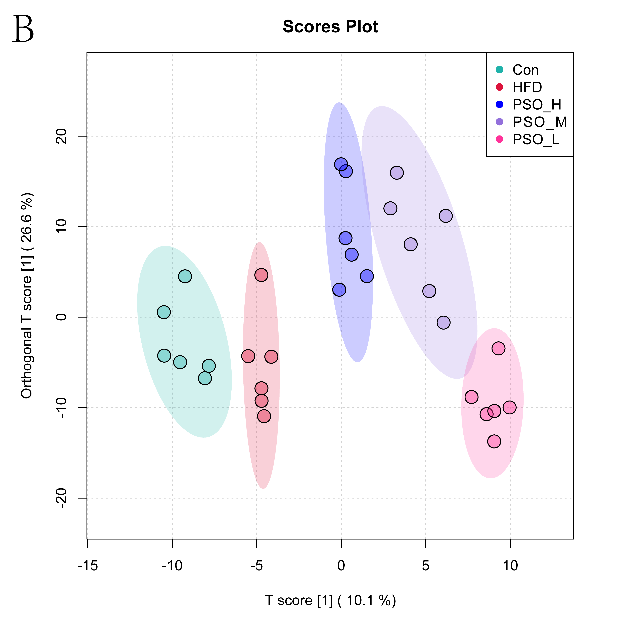


**Figure S3.** PSO modulated the fecal metabolism in negative mode. (A) PLS-DA score plot of Con, HFD, PSO-H, PSO-M and PSO-L groups. (B) OPLS-DA score plot from Con, HFD, PSO-H, PSO-M and PSO-L groups.­


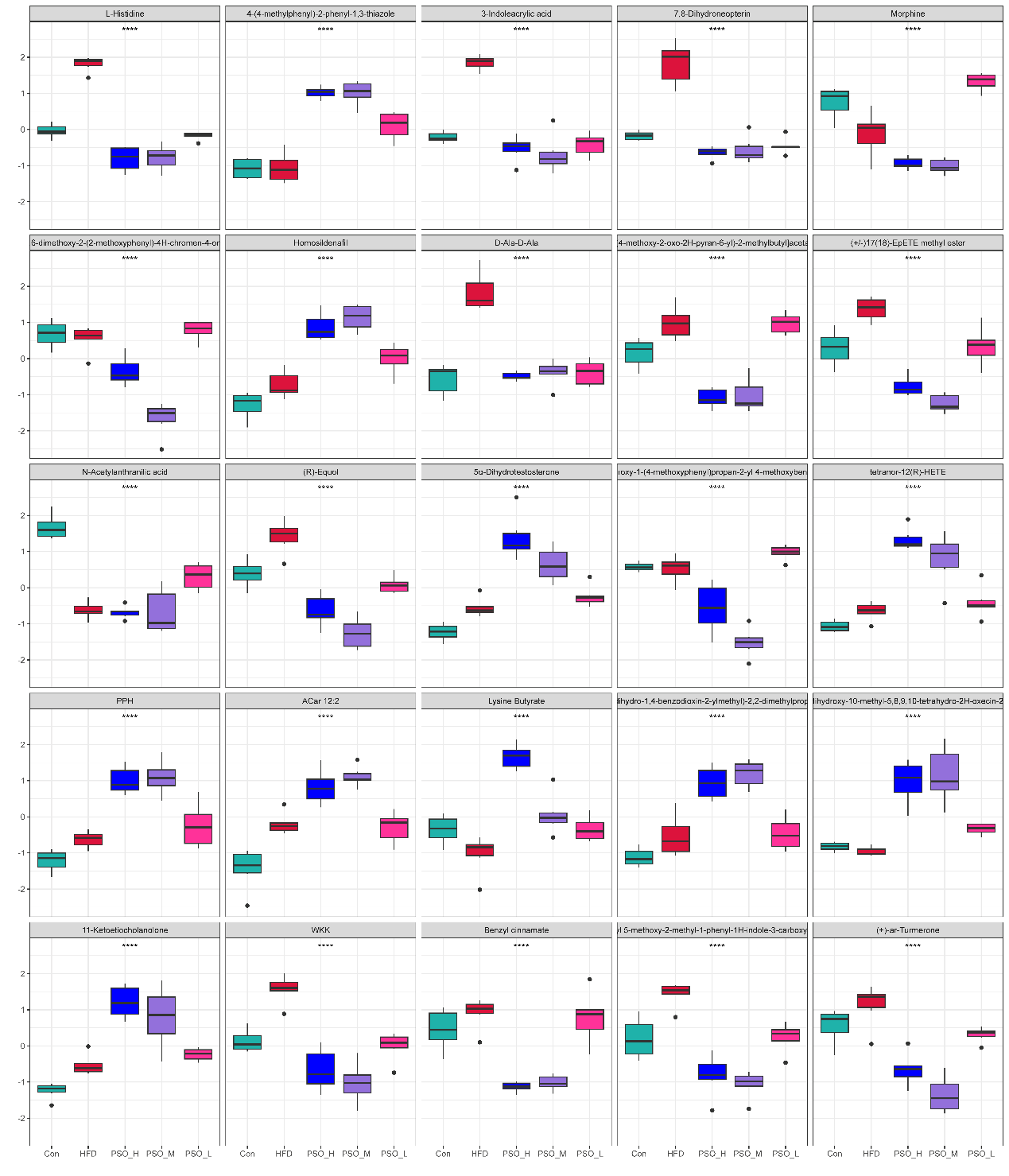


**Figure S4.** Box diagram of metabolite difference (*, **, *** correspond to *P* < 0.05, *P* < 0.01, *P* < 0.001, respectively in positive mode).


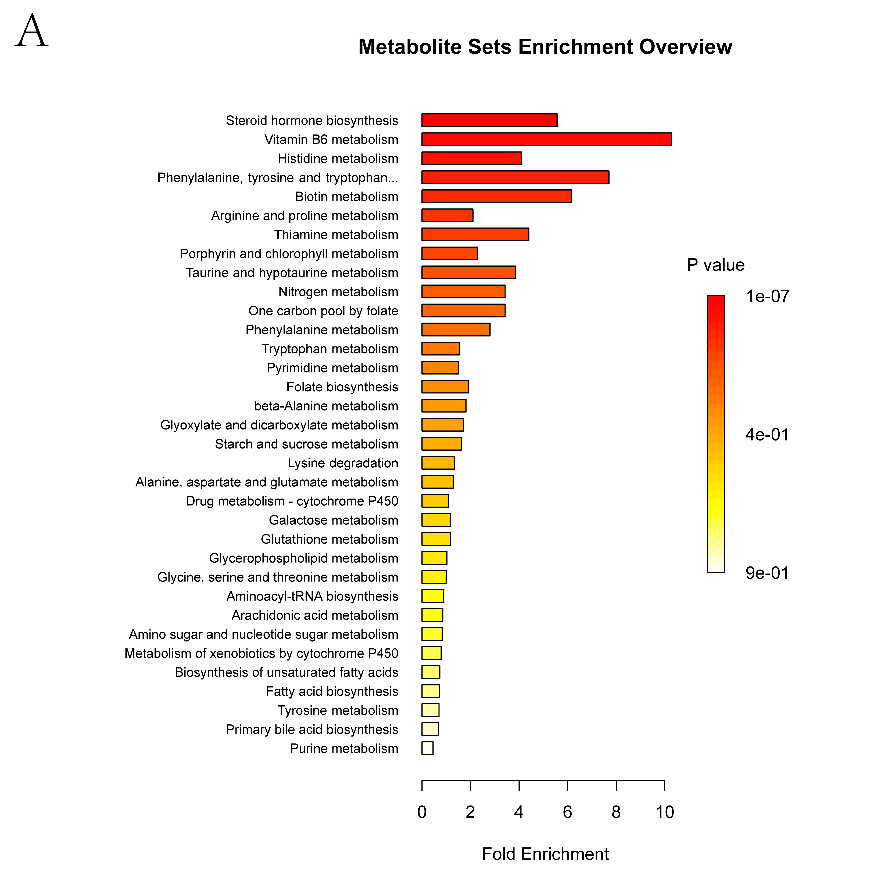


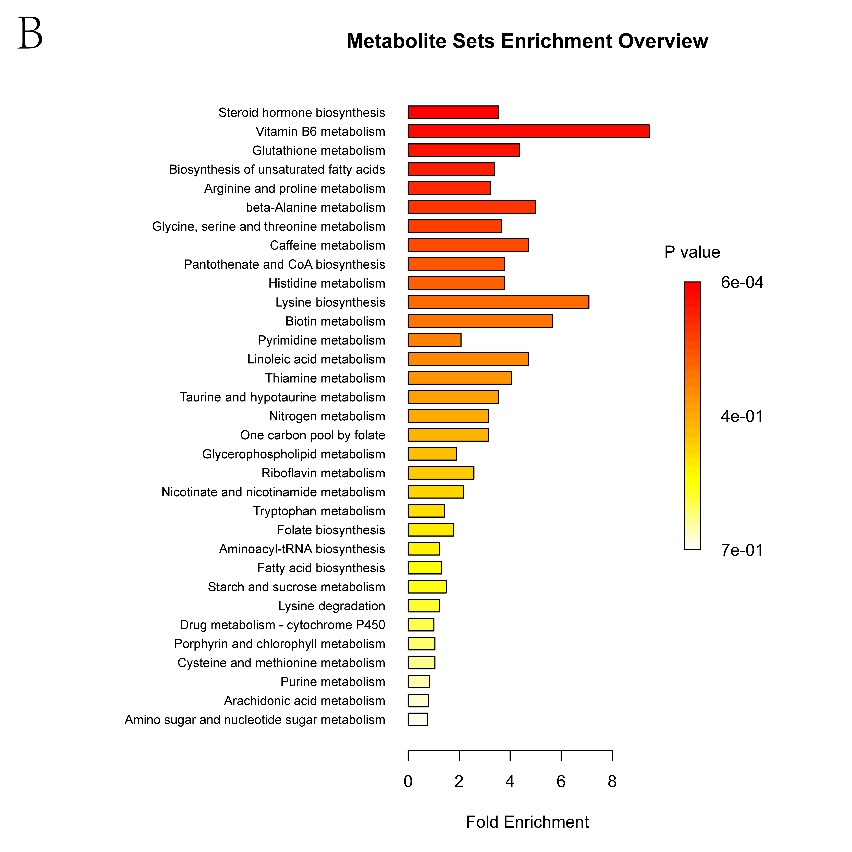


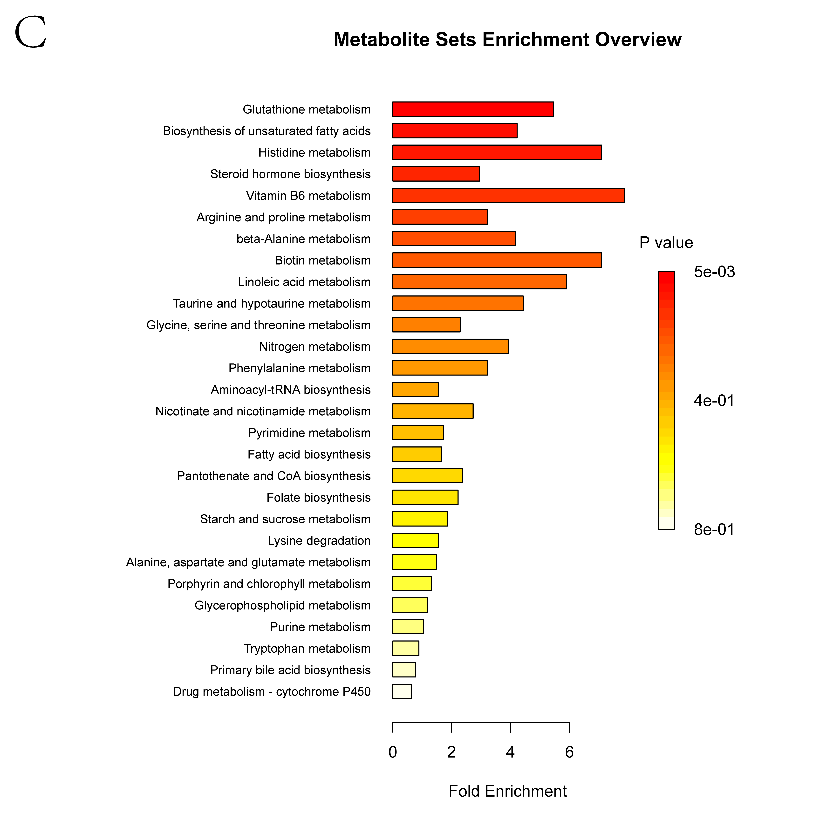


**Figure S5**. Metabolites enrichment pathway analysis of different groups in positive mode: Con vs HFD (A), PSO-M vs HFD (B) and PSO-L vs HFD (C).
