# Supplementary material for: Tree peony seed oil alleviates hyperlipidemia and hyperglycemia by modulating gut microbiota and metabolites in high‐fat diet mice
Source: Food Sci Nutr. 2024 Apr 4;12(6):4421–34. doi: 10.1002/fsn3.4108 (PMC11167153; doi:10.1002/fsn3.4108)
Supplement: Supplementary file 2 — Table S1. [file FSN3-12-4421-s002.docx]

**Tree peony seed oil alleviates hyperlipidemia and hyperglycemia by modulating gut microbiota and metabolites in high-fat diet mice**

**Supplementary Tables**

**Supplementary Table 1.** Information of 16S rRNA sequence from 30 fecal samples.

| Sample | Kingdom | Phylum | Class | Order | Family | Genus | Species | OTU |
| --- | --- | --- | --- | --- | --- | --- | --- | --- |
| Con1 | 628 | 619 | 614 | 586 | 446 | 220 | 59 | 628 |
| Con2 | 602 | 598 | 598 | 562 | 427 | 224 | 60 | 602 |
| Con3 | 675 | 663 | 663 | 643 | 502 | 256 | 66 | 675 |
| Con4 | 569 | 565 | 564 | 552 | 453 | 220 | 69 | 569 |
| Con5 | 670 | 659 | 657 | 639 | 501 | 262 | 70 | 670 |
| Con6 | 895 | 882 | 878 | 844 | 617 | 303 | 71 | 895 |
| HFD1 | 764 | 752 | 747 | 719 | 568 | 274 | 53 | 764 |
| HFD2 | 817 | 806 | 801 | 767 | 592 | 322 | 87 | 817 |
| HFD3 | 686 | 675 | 671 | 636 | 502 | 238 | 71 | 686 |
| HFD4 | 825 | 816 | 812 | 783 | 553 | 266 | 53 | 825 |
| HFD5 | 790 | 781 | 781 | 751 | 554 | 249 | 45 | 790 |
| HFD6 | 801 | 786 | 784 | 752 | 566 | 252 | 59 | 801 |
| PSO-H1 | 831 | 821 | 817 | 781 | 546 | 276 | 63 | 831 |
| PSO-H2 | 711 | 701 | 701 | 676 | 524 | 247 | 48 | 711 |
| PSO-H3 | 797 | 782 | 780 | 761 | 567 | 271 | 59 | 797 |
| PSO-H4 | 691 | 683 | 683 | 663 | 523 | 263 | 60 | 691 |
| PSO-H5 | 861 | 854 | 851 | 828 | 623 | 304 | 61 | 861 |
| PSO-H6 | 532 | 522 | 522 | 506 | 400 | 186 | 41 | 532 |
| PSO-M1 | 435 | 427 | 426 | 408 | 346 | 163 | 41 | 435 |
| PSO-M2 | 383 | 376 | 375 | 369 | 323 | 187 | 48 | 383 |
| PSO-M3 | 273 | 270 | 270 | 265 | 209 | 103 | 24 | 273 |
| PSO-M4 | 317 | 316 | 316 | 310 | 254 | 132 | 32 | 317 |
| PSO-M5 | 333 | 330 | 329 | 322 | 260 | 127 | 24 | 333 |
| PSO-M6 | 309 | 308 | 306 | 301 | 247 | 116 | 25 | 309 |
| PSO-L1 | 372 | 371 | 371 | 366 | 280 | 130 | 25 | 372 |
| PSO-L2 | 407 | 402 | 402 | 393 | 305 | 151 | 29 | 407 |
| PSO-L3 | 355 | 354 | 354 | 340 | 256 | 121 | 20 | 355 |
| PSO-L4 | 300 | 299 | 299 | 297 | 229 | 110 | 25 | 300 |
| PSO-L5 | 560 | 558 | 556 | 532 | 399 | 197 | 42 | 560 |
| PSO-L6 | 498 | 493 | 489 | 464 | 355 | 186 | 27 | 498 |

**Supplementary Table 2.** List of differential fecal metabolites in positive mode.

| No | Metabolite | Formula | Average Molecular Weight | RT [min] | m/z | | HMDB ID | Compound class |
| --- | --- | --- | --- | --- | --- | --- | --- | --- |
| 1 | L-Tyrosine | C_9_ H_11_ NO_3_ | 181.07 | 2.38 | 182.08 | HMDB0000158 | | Organic acids and derivatives |
| 2 | 2-Hydroxycinnamic acid | C_9_ H_8_ O_3_ | 164.04 | 2.39 | 165.05 | HMDB0002641 | | Phenylpropanoids and polyketides |
| 3 | Epinephrine | C_9_ H_13_ NO_3_ | 183.08 | 4.90 | 184.09 | HMDB0000068 | | Benzenoids |
| 4 | isoleucine | C_6_ H_13_ NO_2_ | 131.09 | 3.07 | 132.10 | HMDB0033923 | | Organic acids and derivatives |
| 5 | L-Tryptophan | C_11_ H_12_ N_2_ O_2_ | 204.08 | 5.18 | 205.09 | HMDB0000929 | | Organoheterocyclic compounds |
| 6 | Indole-3-acrylic acid | C_11_ H_9_ NO_2_ | 187.06 | 5.18 | 188.07 | HMDB0000734 | | Organoheterocyclic compounds |
| 7 | Tiglic acid | C_5_ H_8_ O_2_ | 100.05 | 1.39 | 101.05 | HMDB0001470 | | Lipids and lipid-like molecules |
| 8 | DL-Norvaline | C_5_ H_11_ NO_2_ | 117.07 | 1.40 | 118.08 | HMDB0251527 | | Organic acids and derivatives |
| 9 | Hypoxanthine | C_5_ H_4_ N_4_ O | 136.03 | 2.02 | 137.04 | HMDB0000157 | | Organoheterocyclic compounds |
| 10 | Ecgonine methyl ester | C_10_ H_17_ NO_3_ | 199.12 | 6.17 | 200.12 | HMDB0006406 | | Alkaloids and derivatives |
| 11 | Creatine | C_4_ H_9_ N_3_ O_2_ | 131.06 | 1.37 | 132.07 | HMDB0000064 | | Organic acids and derivatives |
| 12 | 19-Nortestosterone | C_18_ H_26_ O_2_ | 274.19 | 7.74 | 275.19 | HMDB0002725 | | Lipids and lipid-like molecules |
| 13 | Glycitein | C_16_ H_12_ O_5_ | 284.06 | 5.95 | 285.07 | HMDB0005781 | | Phenylpropanoids and polyketides |
| 14 | Oleic acid | C_18_ H_34_ O_2_ | 300.26 | 8.76 | 301.27 | HMDB0000207 | | Lipids and lipid-like molecules |
| 15 | 2,4-dihydroxyheptadec-16-en-1-yl acetate | C_19_ H_36_ O_4_ | 310.24 | 8.94 | 311.25 | - | | - |
| 16 | Glycerol 1-hexadecanoate | C_19_ H_38_ O_4_ | 330.27 | 8.44 | 331.28 | HMDB0011564 | | Lipids and lipid-like molecules |
| 17 | 9-Oxo-ODE | C_18_ H_30_ O_3_ | 294.21 | 7.01 | 295.22 | HMDB0004669 | | Lipids and lipid-like molecules |
| 18 | Prostaglandin H1 | C_20_ H_34_ O_5_ | 336.22 | 8.38 | 337.23 | HMDB0013041 | | Lipids and lipid-like molecules |
| 19 | Hexadecanamide | C_16_ H_33_ NO | 255.25 | 9.59 | 256.26 | HMDB0012273 | | Lipids and lipid-like molecules |
| 20 | Oleamide | C_18_ H_35_ NO | 281.27 | 9.80 | 282.27 | HMDB0002117 | | Lipids and lipid-like molecules |
| 21 | 3-Oxo-7alpha,12alpha-hydroxy-5beta-cholanoic acid | C_24_ H_38_ O_5_ | 406.27 | 6.88 | 407.27 | - | | - |
| 22 | MAG (18:3) | C_21_ H_36_ O_4_ | 352.25 | 7.45 | 353.26 | - | | - |
| 23 | Sedanolide | C_12_ H_18_ O_2_ | 176.11 | 6.82 | 177.12 | HMDB0302242 | | Organoheterocyclic compounds |
| 24 | Monoolein | C_21_ H_40_ O_4_ | 356.29 | 9.83 | 357.29 | HMDB0254854 | | Lipids and lipid-like molecules |
| 25 | 7-Ketocholesterol | C_27_ H_44_ O_2_ | 400.33 | 9.86 | 401.34 | HMDB0000501 | | Lipids and lipid-like molecules |
| 26 | TKK | C_16_ H_33_ N_5_ O_5_ | 375.25 | 6.59 | 376.25 | - | | - |
| 27 | Nicotinic acid | C_6_ H_5_ NO_2_ | 123.03 | 1.83 | 124.03 | HMDB0001488 | | Organoheterocyclic compounds |
| 28 | Palmitoyl ethanolamide | C_18_ H_37_ NO_2_ | 299.28 | 9.50 | 300.28 | HMDB0002100 | | Organic acids and derivatives |
| 29 | Linoleoyl ethanolamide | C_20_ H_37_ NO_2_ | 323.28 | 9.18 | 324.28 | HMDB0012252 | | Organic nitrogen compounds |
| 30 | (-)-Caryophyllene oxide | C_15_ H_24_ O | 220.18 | 8.75 | 221.18 | - | | - |

**Supplementary Table 3.** List of differential fecal metabolites in negative mode.

| No | Metabolite | Formula | Average Molecular Weight | RT [min] | m/z | | HMDB ID | Compound class |
| --- | --- | --- | --- | --- | --- | --- | --- | --- |
| 1 | 13-Hpotre(R) | C_18_ H_30_ O_4_ | 310.21 | 7.33 | 309.20 | - | | - |
| 2 | 13(S)-HOTrE | C_18_ H_30_ O_3_ | 294.21 | 8.03 | 293.21 | - | | - |
| 3 | (±)9-HpODE | C_18_ H_32_ O_4_ | 312.23 | 6.97 | 311.22 | - | | - |
| 4 | Gamma-Nonanolactone | C_9_ H_16_ O_2_ | 156.11 | 7.27 | 155.10 | - | | - |
| 5 | Corchorifatty acid F | C_18_ H_32_ O_5_ | 328.22 | 6.60 | 327.21 | HMDB0035919 | | Lipids and lipid-like molecules |
| 6 | 3,8,9-trihydroxy-10-propyl-3,4,5,8,9,10-hexahydro-2H-oxecin-2-one | C_12_ H_20_ O_5_ | 244.13 | 5.90 | 243.12 | - | | - |
| 7 | 2,3-dinor Prostaglandin E1 | C_18_ H_30_ O_5_ | 326.20 | 6.59 | 325.20 | - | | - |
| 8 | Azelaic acid | C_9_ H_16_ O_4_ | 188.10 | 5.96 | 187.09 | HMDB0000784 | | Lipids and lipid-like molecules |
| 9 | (3-Methoxy-4-hydroxyphenyl)ethylene glycol sulfate | C_9_ H_12_ O_7_ S | 264.03 | 5.51 | 263.02 | HMDB0000559 | | Organic acids and derivatives |
| 10 | N'-(4-chlorophenyl)-4-ethylbenzohydrazide | C_15_ H_15_ Cl N_2_ O | 274.08 | 7.39 | 273.08 | - | | - |
| 11 | Phloretin | C_15_ H_14_ O_5_ | 274.08 | 7.26 | 273.08 | HMDB0003306 | | Phenylpropanoids and polyketides |
| 12 | Catechin | C_15_ H_14_ O_6_ | 290.08 | 5.69 | 289.07 | HMDB0002780 | | Phenylpropanoids and polyketides |
| 13 | Salvinorin B | C_21_ H_26_ O_7_ | 390.17 | 6.87 | 389.16 | - | | - |
| 14 | Cholic acid | C_24_ H_40_ O_5_ | 408.28 | 7.47 | 407.28 | HMDB0000619 | | Lipids and lipid-like molecules |
| 15 | 7-Hydroxy-3,4-dihydrocarbostyril | C_9_ H_9_ N O_2_ | 163.06 | 5.36 | 162.05 | - | | - |
| 16 | DL-m-Tyrosine | C_9_ H_11_ N O_3_ | 181.07 | 5.35 | 180.06 | - | | - |
| 17 | Dodecanedioic acid | C_12_ H_22_ O_4_ | 230.15 | 6.62 | 229.14 | HMDB0000623 | | Lipids and lipid-like molecules |
| 18 | Elaidic acid | C_18_ H_34_ O_2_ | 282.25 | 10.50 | 281.24 | HMDB0000573 | | Lipids and lipid-like molecules |
| 19 | N-Acetyl-DL-glutamic acid | C_7_ H_11_ N O_5_ | 189.06 | 2.27 | 188.05 | - | | - |
| 20 | L-Glutamate | C_5_ H_9_ N O_4_ | 147.05 | 1.47 | 146.04 | HMDB0060475 | | Organic acids and derivatives |
| 21 | LPC 16:0 | C_24_ H_50_ N O_7_ P | 541.33 | 9.38 | 540.33 | - | | - |
| 22 | Arachidonic acid | C_20_ H_32_ O_2_ | 304.24 | 9.85 | 303.23 | HMDB0001043 | | Lipids and lipid-like molecules |
| 23 | 7-Ketolithocholic acid | C_24_ H_38_ O_4_ | 390.27 | 7.84 | 389.27 | HMDB0000467 | | Lipids and lipid-like molecules |
| 24 | Deoxycholic acid | C_24_ H_40_ O_4_ | 392.29 | 8.19 | 391.28 | HMDB0000626 | | Lipids and lipid-like molecules |
| 25 | 3-Phenyllactic acid | C_9_ H_10_ O_3_ | 166.06 | 5.77 | 165.05 | HMDB0000779 | | Phenylpropanoids and polyketides |
| 26 | (S)-Leucic acid | C_6_ H_12_ O_3_ | 132.07 | 5.75 | 131.07 | HMDB0000746 | | Lipids and lipid-like molecules |
| 27 | Phenylacetaldehyde | C_8_ H_8_ O | 120.05 | 5.67 | 119.05 | HMDB0006236 | | Benzenoids |
| 28 | (±)9(10)-DiHOME | C_18_ H_34_ O_4_ | 314.24 | 7.15 | 313.23 | - | | - |
| 29 | (+/-)12(13)-DiHOME | C_18_ H_34_ O_4_ | 296.23 | 8.06 | 295.22 | - | | - |
| 30 | (+/-)9-HpODE | C_18_ H_32_ O_4_ | 312.23 | 7.41 | 311.22 | - | | - |
